# The PanOryza pangene catalog of Asian cultivated rice

**DOI:** 10.1101/gr.280790.125

**Published:** 2026-01

**Authors:** Bruno Contreras-Moreira, Eshan Sharma, Shradha Saraf, Guy Naamati, Parul Gupta, Justin Elser, Dmytro Chebotarov, Kapeel Chougule, Zhenyuan Lu, Sharon Wei, Andrew Olson, Ian Tsang, Disha Lodha, Yong Zhou, Zhichao Yu, Wen Zhao, Jianwei Zhang, Sandeep Amberkar, Kawinnat Sue-Ob, Zhi Sun, Maria Martin, Kenneth L. McNally, Doreen Ware, Eric W. Deutsch, Dario Copetti, Rod A. Wing, Pankaj Jaiswal, Sarah Dyer, Andrew R. Jones

**Affiliations:** 1 Department of Genetics and Plant Breeding, Estación Experimental de Aula Dei–Consejo Superior de Investigaciones Científicas, 50059, Zaragoza, Spain;; 2 European Molecular Biology Laboratory, European Bioinformatics Institute, Wellcome Genome Campus, Hinxton CB10 1SD, United Kingdom;; 3 University of Liverpool, Institute of Systems, Molecular and Integrative Biology, Liverpool L69 7ZB, United Kingdom;; 4 Department of Botany and Plant Pathology, Oregon State University, Corvallis, Oregon 97331, USA;; 5 International Rice Research Institute (IRRI), Los Baños, 4031 Laguna, Philippines;; 6 Cold Spring Harbor Laboratory, Cold Spring Harbor, New York 11724, USA;; 7 NIAB, Cambridge CB3 0LE, United Kingdom;; 8 University of Nottingham, Department of Plant Science, Nottingham LE12 5RD, United Kingdom;; 9 Biological and Environmental Sciences and Engineering Division (BESE), King Abdullah University of Science and Technology (KAUST), Thuwal, 23955-6900, Saudi Arabia;; 10 National Key Laboratory of Crop Genetic Improvement, Huazhong Agricultural University, Wuhan 430070, China;; 11 Institute for Systems Biology, Seattle, Washington 98109, USA;; 12 USDA ARS NEA Plant, Soil & Nutrition Laboratory Research Unit, Ithaca, New York 14853, USA;; 13 Arizona Genomics Institute, School of Plant Sciences, The University of Arizona, Tucson, Arizona 85721, USA

## Abstract

The rice genome underpins fundamental research and breeding, but the Nipponbare (*japonica*) reference does not fully encompass the genetic diversity of Asian rice. To address this gap, the Rice Population Reference Panel (RPRP) was developed, comprising high-quality assemblies of 16 rice cultivars to represent the *japonica*, *indica*, *aus*, and *aromatic* varietal groups. The RPRP has been consistently annotated and supported by extensive experimental data, and here, we report the computational assignment, characterization, and dissemination of stably identified pangenes, collectively called the PanOryza data set. We identify 25,178 core pangenes shared across all cultivars, alongside cultivar-specific and family-enriched genes. Core genes exhibit higher gene expression and proteomic evidence, higher confidence protein domains, and AlphaFold structures, whereas cultivar-specific genes are enriched for domains under selective breeding pressure, such as for disease resistance. We identify more than 5000 genes absent in the IRGSP rice reference genome and present in at least two other *Oryza* cultivars. We demonstrate the utility of this resource through various examples of pangenes and their protein domains. This resource, integrated into public databases, enables researchers to explore genetic and functional diversity via a population-aware “reference guide” across rice genomes, advancing both basic and applied research.

Rice is one of the most important crops for human nutrition and will be central to efforts to feed 9.8 billion people by 2050. Breeding efforts are targeted with increasing yields and nutrition, while also making rice more resistant to biotic and abiotic stresses, which will be exacerbated by a changing climate. Asian cultivated rice *Orzya sativa* L. has previously been classified in different varietal groups, based on historic domestication events, mostly notably *indica* (*Xian*) and *japonica* (*Geng*), with other recognized groups including those described as *aus*, *aromatic* (Basmati), and *admixed* (Wang et al. 2018).

An important resource underpinning both basic and applied research is the rice reference genome sequence, originally released with two draft assemblies circa 2002 (for *indica* and *japonica*) (Goff et al. 2002; Yu et al. 2002) and then with one finished genome 3 years later by the International Rice Genome Sequencing Project (IRGSP), with an annotation set (gene models, gene, and protein sequences) released in 2005 (International Rice Genome Sequencing Project and Sasaki 2005). The IRGSP canonical reference (IRGSP RefSeq) genome is derived from the *japonica* variety Nipponbare, despite *indica* rice accounting for a much larger share of the international market. In the past 20 years, there have been multiple efforts to annotate the IRGSP reference, using different software packages and supporting data. From these efforts, two primary annotation sets have persisted: those developed by the Rice Annotation Project Database (RAP-DB; example gene identifier, Os01g0918300) (Sakai et al. 2013) and the Rice Genome Annotation Project at Michigan State University (MSU; example, LOC_Os01g68950) (Ouyang et al. 2007). RAP-DB is still actively updating its database, whereas the MSU annotations have been frozen since 2014, with the latter being the more widely used/reference annotations (for historical reasons).

To study genetic variation, significant efforts have been made to identify variants on a large scale from high-throughput short DNA read sequencing, for example, via the 3000 Rice Genomes Project (Li et al. 2014; Wang et al. 2018). These projects can identify single-nucleotide polymorphisms (SNPs) or short insertions and deletions, relative to the reference genome to which they are mapped. However, they cannot be used to find larger structural rearrangements of chromosomes, to call variants relative to any regions absent from the reference genome used, or to define accurate gene models for regions of chromosomes absent from the reference genome.

Fully assembled genomes allow researchers to study chromosomal rearrangements and potentially identify gene gain or loss in different rice varieties. A collection of “platinum-standard” genomes assemblies, called the Rice Population Reference Panel (RPRP; also called the “MAGIC-16”) (Zhang et al. 2016; Zhou et al. 2020), has been created, including 15 new genomes (plus the IRGSP RefSeq) aimed specifically at covering a significant portion of the population genetic diversity of *Oryza sativa*. These genomes have now been annotated using a consistent pipeline (for description, see Zhou et al. 2020), including support from long-read transcriptome data in every variety, which is able to give strong experimental evidence for the correct splicing prediction of gene models. As new genomes are sequenced, assembled, and annotated, they have the potential to act as a powerful resource for rice researchers, for example, to find genes/variants present in only certain varieties or where genes are differentially alternatively spliced across varieties. However, there are currently no stable/consistent nomenclature gene identifiers across the rice pangenome, and it is not straightforward to determine the relationships between orthologous genes.

In this work, we built a stable pangene set for Asian rice (*O. sativa*), based on whole-genome alignment (WGA) of 16 very high-quality assemblies and gene annotation sets, supported by multiomic data sets. We identify syntenic orthologs across the annotated RPRP genomes using the GET_PANGENES algorithm we developed based on WGA (Contreras-Moreira et al. 2023). Further, we assigned stable identifiers for a *pangene* set, here defined as a collection of gene models across different varieties in the same genomic location, following the WGA. Having defined pangenes, we next explore their characteristics with respect to their occupancy across genomes and the extent to which their gene expression and protein abundance is supported by experimental data from multiple sources. We explore protein domains for their occupancy across pangene groups with an aim to identify candidate pangenes for functional characterization and their utilization toward rice improvement. Lastly, a key objective of the study is to allow straightforward user access to all gene annotation sets or pangene identifiers for easy exploration in a longer term, allowing updates to the pangenome resources. Therefore, these resources have been made available through end-user focused databases; Ensembl Plants, Gramene, and protein sequences have been deposited in UniProtKB.

## Results

### RPRP gene sets

We first collated gene models for the RPRP genomes as a source for the creation of a pan-*Oryza* gene set. All 16 genomes, including the IRGSP RefSeq were annotated using a consistent pipeline and data source (see Methods). In addition, there are also RAP-DB and MSU gene sets for the IRGSP RefSeq, making 18 annotation sets in total from which we built the pangene set. The counts of gene models for each genome used as input to the pangene set are provided in Table 1. In each accession, around 36,000 genes were identified except for Nipponbare, which has a greatly inflated gene count owing to the merging of three input sets (see “nipponbare_merged_GFF” at https://zenodo.org/records/14772953).

**Table 1.** Summary information on the RPRP genomes and their annotations

Stock accession^a^	RPRP gene model prefix	Genetic stock varname	Country origin	K15 subpops	K15 common	K5 groups	Gene count
IRGC 117425	OsARC_	ARC 10497::IRGC 12485-1	India	cB	cBasmati	*aro*	36,442
IRGC 117264	OsAzu_	Azucena	Philippines	GJ-trop1	tropA	Tropjap	36,626
IRGC 132278	OsCMeo_	CHAO MEO::IRGC 80273-1	Lao PDR	GJ-subtrp	subtrop	Tropjap	36,999
IRGC 132424	OsGoSa_	GOBOL SAIL (BALAM)::IRGC 26624-2	Bangladesh	XI-2A	ind2A	*indica*	36,237
IRGC 117268	OsIR64_	IR 64	Philippines	XI-1B1	ind1B1	*indica*	36,102
IRGC 128077	OsKeNa_	KETAN NANGKA::IRGC 19961-2	Indonesia	GJ-trop2	tropB	Tropjap	36,612
IRGC 127518	OsKYG_	KHAO YAI GUANG::IRGC 65972-1	Thailand	XI-3B1	ind3B1	*indica*	36,476
IRGC 125619	OsLaMu_	LARHA MUGAD::IRGC 52339-1	India	XI-2B	ind2B	*indica*	36,316
IRGC 127564	OsLima_	LIMA::IRGC 81487-1	Indonesia	XI-3A	ind3A	*indica*	37,044
IRGC 125827	OsLiXu_	LIU XU::IRGC 109232-1	China	XI-3B2	ind3B2	*indica*	36,378
	OsMH63_	Minghui 63	China	XI-adm	ind-admx	*indica*	36,147
IRGC 117534	OsN22_	N22::IRGC 19379-1	India	cA1	cAus1 (Assam)	*aus*	36,319
IRGC 127652	OsNaBo_	NATEL BORO::IRGC 34749-1	Bangladesh	CA2	cAus2 (Bengal)	*aus*	36,196
IRGC 117274	Os (RAP-DB); Loc_Os (MSU); Gramene annotations OsNip_	Nipponbare (IRGSP1.0)	Japan	GJ-temp	temp	Tempjap	62,225^b^
IRGC 127742	OsPr106_	PR 106::IRGC 53418-1	India	XI-1B2	ind1B2	*indica*	36,436
IRGC 117280	OsZS97_	Zhen Shan 97	China	XI-1A	ind1A	*indica*	35,686

^a^Stock accession or closest OryzaSNP_accession.

^b^Breakdown by source prior to merging: RAP-DB = 35,694, MSU = 55,801 (including possible transposable elements), OsNip = 37,007.

### Pangene sets, clusters, and identifiers

**Figure 1.**
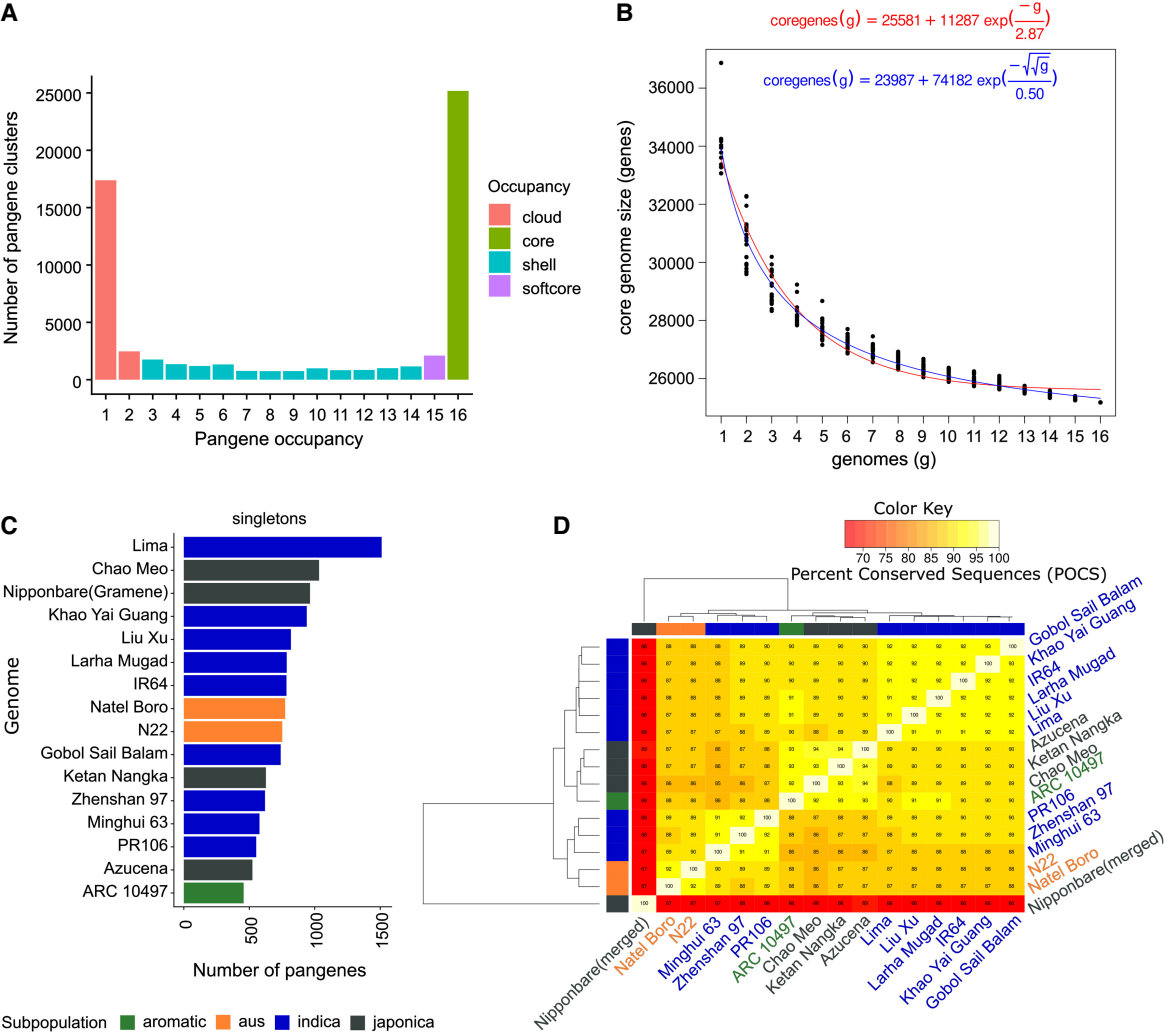
Pangene sets of rice. (*A*) Count of input genome members (*x*-axis) “occupancy” 1–16 versus counts of pangenes (pangenes containing only one MSU gene model have been removed). (*B*) Core-genome growth simulations after adding RPRP cultivars in random order (without singleton genes) using Tettelin (red) (Tettelin et al. 2005) and Willenbrock (blue) (Willenbrock et al. 2007) functions, fitted after 20 permutation experiments. (*C*) Counts of singletons, that is, genes per input genome present in clusters with no other gene models (for IRGSP, singleton count includes those with an OsNip [Gramene-generated] gene, for unbiased comparison). (*D*) Matrix of the percentage of shared clusters and heat map of RPRP pangenes. The dendrograms were computed by complete linkage clustering and Euclidean distances computed among columns.

Our first objective was to analyze gene annotation sets from the RPRP genomes and assign pangenes following WGA, indicative of synteny. The GET_PANGENES pipeline was used to create pangenes, determine their occupancy statistics, and assign long-term stable identifiers (Fig. 1). Supplemental Table S1 contains the matrix of pangenes, with their stable identifier (column 1) and then 16 further columns, one per input genome, containing transcript identifiers (if any) from each genome that have been mapped to that pangene. There are a total of 77,530 pangenes, including 35,029 singletons, that is, pangenes containing transcripts from only one genome (17,383 singletons when MSU gene models are removed, which heavily inflate the count), leaving 42,501 pangene clusters containing two or more members. Figure 1A displays the distribution of pangenes by occupancy class, demonstrating that most genes are core or cloud and that shell genes are relatively rare in the pangene set.

We also assessed the quality and robustness of pangene formation through multiple metrics, including descriptive statistics on gene length, exon count, sequence distance between members, and “MSA completeness” (Supplemental Fig. S1; Supplemental Table S2), demonstrating that the majority of pangenes contain highly similar gene/protein sequences from different genomes. The metrics also allow for identification of pangenes containing one or more inconsistent members, indicative of different overlapping gene predictions in some genomes (e.g., coding sequences predicted on different frames), which can be artifacts of incorrect gene model prediction.

Figure 1B shows that the core set of genes converge at 25,178 genes according to the Tettelin method (23,987 by Willenbrock method), indicating this is the minimal core set contained within an *O. sativa* genome. The pangene curve shows that the pangenome size starts converging at around 42,000 genes (Supplemental Fig. S2). Figure 1C displays the counts per variety of singletons from each genome, ranging from 457 to 1511 (mean, 766; median, 752), indicating consistency across different cultivars for the presence of these unique genes. For Nipponbare, the results are filtered to include singletons containing an OsNip-source model, created using the same approach as the other 15 genomes to avoid artefactually high counts owing to merging different input gene sets, giving a set of 964 genes in line with other genomes. Figure 1D shows the pangene cluster overlap across cultivars. In general, there is reasonable clustering by rice subfamily (*japonica* vs. *aus* vs. *indica*). The merged Nipponbare annotation has lower overlap with other clusters, owing to it having a much higher count of input genes.

### Genomic positions of pangenes

The GET_PANGENES approach uses WGA and does not enforce that genes must be located on the same chromosome. This enables the algorithm to identify cases in which breeding (or natural selection) has caused regions of the chromosome to translocate or potentially in which there have been assembly errors. In Supplemental Figure S3, we display the chromosomal location of genes within the pangene set against the Nipponbare (IRGSP) reference genome (see Methods). There are sporadic cases of genes that are not collinear with respect to the reference genome, with a few notable systematic cases. These include a set of genes that are inverted on Chromosome 1 for OsLima at position 600,000–800,000 bp with respect to all other genomes. Similarly, there are two inversions on *aus* genomes (N22 and Natal Boro) around the center of Chromosome 1. Another notable case is present on Chromosome 6, in which Nipponbare has a large inversion compared with the other 15 genomes. Data containing the positions of genes within pangene clusters can be found in Supplemental Table S3. There are 1502 pangenes that have occupancy greater than two and contain genes mapped to different chromosomes (Supplemental Table S3), which researchers should consider when using genomics resources, for example, SNP mapping or allele mining.

### Pangene distribution across rice families

Supplemental Table S4 contains the proportions of genomes by subfamily (*japonica*, *aromatic*, *aus*, *indica*) that have a member within each pangene. For example, by filtering these data, one can identify genes that segregate by family, for example, 359 pangenes found in all *japonica* genomes but only zero or one other genome, 229 pangenes found in eight or nine *indica* genomes but present in zero or one other genomes, and 336 pangenes found in *aus* varieties and zero or one other genomes. For multiple research and breeding purposes, such genes are of high interest, particularly if there are QTLs mapped to these genomic regions. One of the current major limitations of genome-wide association or other population SNP mapping studies is that the ISRFG RefSeq reference genome (IRGSP) is almost always used for read mapping and locating genes near SNPs. Unfortunately, as we show here, there are 17,130 pangenes that do not have an IRGSP gene model (5634 with occupancy of two or more), indicative of a large pool of genetic resources that is currently missed in most such studies.

### Properties of pangenes by occupancy

**Figure 2.**
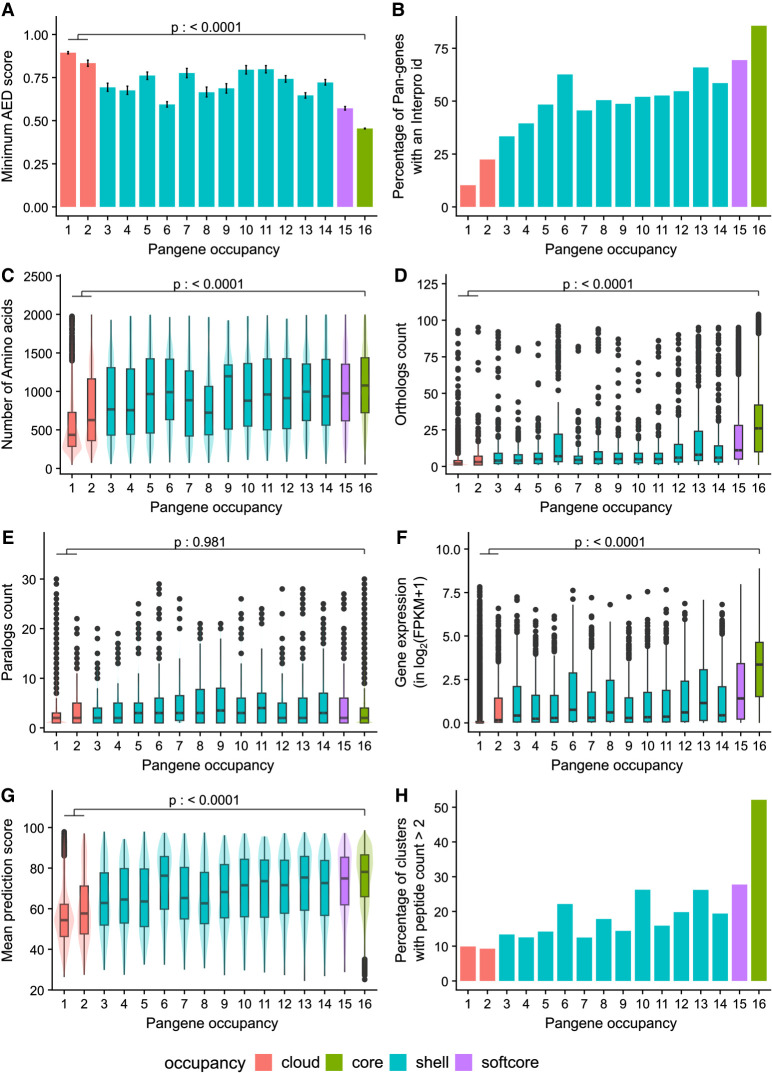
Exploration of pangenes and their properties by their occupancy. The *x*-axis shows the pangene occupancy for each cluster; the *y*-axis shows data derived from RAP-DB gene models within those clusters (clusters containing no RAP-DB model are excluded). (*A*) Mean of minimum AED score from three transcriptomic data sets. (*B*) Proportion of genes that contain a significant match to an InterPro domain. (*C*) Mean count of amino acids. (*D*) Boxplot showing the counts of orthologs derived from Ensembl Compara (see Methods). (*E*) Boxplot showing counts of paralogs. (*F*) Boxplot of mean quantitative gene expression data log_2_(FPKM + 1), sourced from 11,726 rice RNA-seq samples. (*G*) Boxplot of mean prediction score (pLDDT) from the corresponding AlphaFold2 model. (*H*) Percentage of clusters with genes having peptide evidence (n > 2 per gene). The *p*-value indicates result from Tukey's HSD test for core versus cloud pangenes.

We next explored the properties of pangenes related to occupancy (Fig. 2). We chose to focus on quality metrics derived from the RAP-DB reference gene annotations (for which most data already exist from different resources) and take the occupancy statistic from the parent pangene. First, we assessed the quality of a given gene model, using the TRaCE (Olson and Ware 2021) algorithm, and assigned a minimum annotation edit distance (AED) score, from three transcriptomic data sets, to assess the quality of the annotation. AED is calculated on a scale of zero to one based on the sequence distance between the gene model and the best transcript, assembled from publicly available transcriptomics data.

Figure 2A shows the mean (minimum) AED score for all RAP-DB genes, against the occupancy of their parent pangene. Core (occupancy, 16) or softcore (15 or 16) clusters have significantly lower AED within core (mean, 0.5) versus cloud pangenes (mean, 0.9), indicating that core genes are called with higher confidence using transcriptomic data sources. Of the core pangene, 83% have an assigned InterPro domain (Fig. 2B), compared with 11% in singleton pangenes (occupancy, 1). A more detailed analysis of protein domains across all pangenes is provided below. Core pangenes are also significantly longer and are more likely conserved across the plant kingdom as indicated by their protein length and ortholog count (Fig. 2C,D).

There is a more complex trend for paralog count (Fig. 2E), in which there is no significant difference between paralog counts for cloud or shell versus core sets. There appears to be a trend in which shell genes have higher paralog counts. We hypothesize that there are two “competing” distributions here. We would expect that pangenes of lower occupancy are more likely to have paralogs, as this set contains rapidly duplicating gene families (further discussed below), but are also enriched for faulty gene models in which paralogs cannot be identified.

Pangenes with softcore/core occupancy have significantly higher gene expression values compared with cloud genes, with singleton pangenes (occupancy, 1) frequently having no detectable signals; these could be pseudogenes or genes only transcribed under particular conditions not assessed in the source data (Fig. 2F). Prediction of a 3D protein structure for core/softcore and shell pangenes can be done by AlphaFold with high confidence (median ± interquartile range: 78.1 ± 20.6 for core, 75.0 ± 23.4 for softcore, and 70.3 ± 27.2 for shell pangenes), whereas a protein structure for cloud pangenes has a median score of 54.7 ± 16.6, indicating lower confidence (Fig. 2G). Cloud genes thus appear to be enriched with low-quality gene models (short sequences, no InterPro domains, weaker structure models), which is further supported by peptide-based evidence derived from experimental proteomics studies (further details below), in which ∼9.9% of singleton pangenes are supported by more than two peptides per gene. In contrast, 52.1% core RAP-DB genes show more than two peptides per gene as evidenced for the pangene cluster (Fig. 2H).

### Support for rice panproteome

To determine experimental support for the rice pangenes, we performed large-scale reanalysis of public proteomics (mass spectrometry) data, against a comprehensive protein database derived from all possible gene models within the pangene set. We searched 19 public data sets (about 30 million MS/MS spectra) sourced and pooled from multiple rice varieties to maximize total coverage. All data sets were from “shotgun” methods; that is, proteins were digested into a total peptide pool prior to LC-MS/MS. As a result, for peptides that can match to more than one gene product (per genome), it is not straightforward to determine which is the correct assignment. Using an all peptide-to-protein mapping, we demonstrate potential peptide-based evidence using more than 200,000 distinct peptides for 435,196 gene models and 293,844 genes present in 28,658 pangenes.

**Figure 3.**
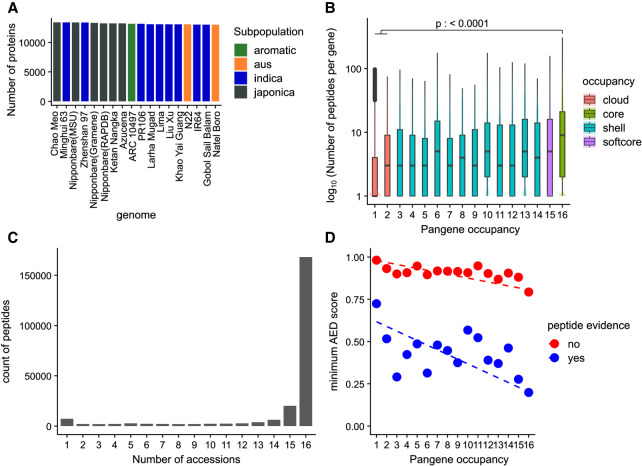
Experimental proteomics data support for pangenes. (*A*) Count of proteins per annotation set with at least two supporting peptides; (*B*) log counts of peptide sequences versus genome occupancy; (*C*) absolute counts of peptides mapped to proteins from one to 16 genomes; and (*D*) scatterplot of mean (min) AED score across three transcriptomics data sets for those (RAP-DB) gene products with or without peptide evidence, by genome occupancy of the pangenes. Dashed line indicates linear regression, indicating that gene products with peptide evidence have better (lower AED) support from transcriptomics data. The *p*-value indicates result from Tukey's HSD test for core versus cloud pangenes.

Taking a parsimonious approach in which a peptide can only be mapped to one protein, we found around 13,000 genes in each genome with at least two peptides, indicating strong evidence for the gene's protein-coding potential (Fig. 3A). In agreement with the previous set of observations, we find a significantly higher number of peptides mapped to core and soft-core pangenes than to cloud pangenes (Fig. 3B). Out of all the peptides mapped to all the proteins, ∼73% (n = 168,151) of the peptides mapped to proteins in all 16 rice accessions, indicating good support for these pangenes, and in general, based on current evidence, there is little deviation in sequences for readily detectable rice proteins across the pangenome (Fig. 3C).

We further demonstrate the relationship between proteomic evidence (those genes with or without proteomics evidence) and AED score (derived from transcriptomes mapped to the RAP-DB gene representative of each pangene cluster) in Figure 3D. Proteins without experimental support tend to have high AED scores (ranging from one to 0.8, depending on genomes occupancy), whereas those with experimental support and high occupancy have low AED scores, indicative of well-supported, high-confidence gene models.

### Exploring the predicted protein domains of pangenes

We ran InterProScan (Jones et al. 2014) over all input proteins available within the pangene set, assigning Pfam (Mistry et al. 2021) and InterPro (Paysan-Lafosse et al. 2023) domains. For each pangene, we assigned additional data types and statistics, including the total number of different domains identified, the genomes (and proteins) containing those domains, and a representative domain for the cluster (most common across genomes), available in Supplemental Table S5. We then assigned which proteins within a pangene cluster contain the representative domain, and which proteins are outliers, that is, do not contain the most common domain. Of the 42,501 pangenes with at least two members, 26,044 have at least one assigned Pfam domain (and 28,507 at least one InterPro domain). Of these, 21,779 are “domain-consistent” clusters (83%), having zero genomes lacking the most common Pfam domain. Similarly, 88% of pangene clusters have a consistent common InterPro domain. In Supplemental Table S6, we show the counts of proteins that do not contain the most common InterPro domain per cluster per input genome for the 3485 pangenes that are not “domain consistent.” There are around 400–600 proteins per input annotation set that do not match the most common domain. RAP-DB and MSU gene models have higher counts but have not been annotated using the same pipeline as other genomes and thus are less consistent than OsNip models compared with other genomes, and we cannot straightforwardly conclude that the gene models from these sources are less reliable.

**Figure 4.**
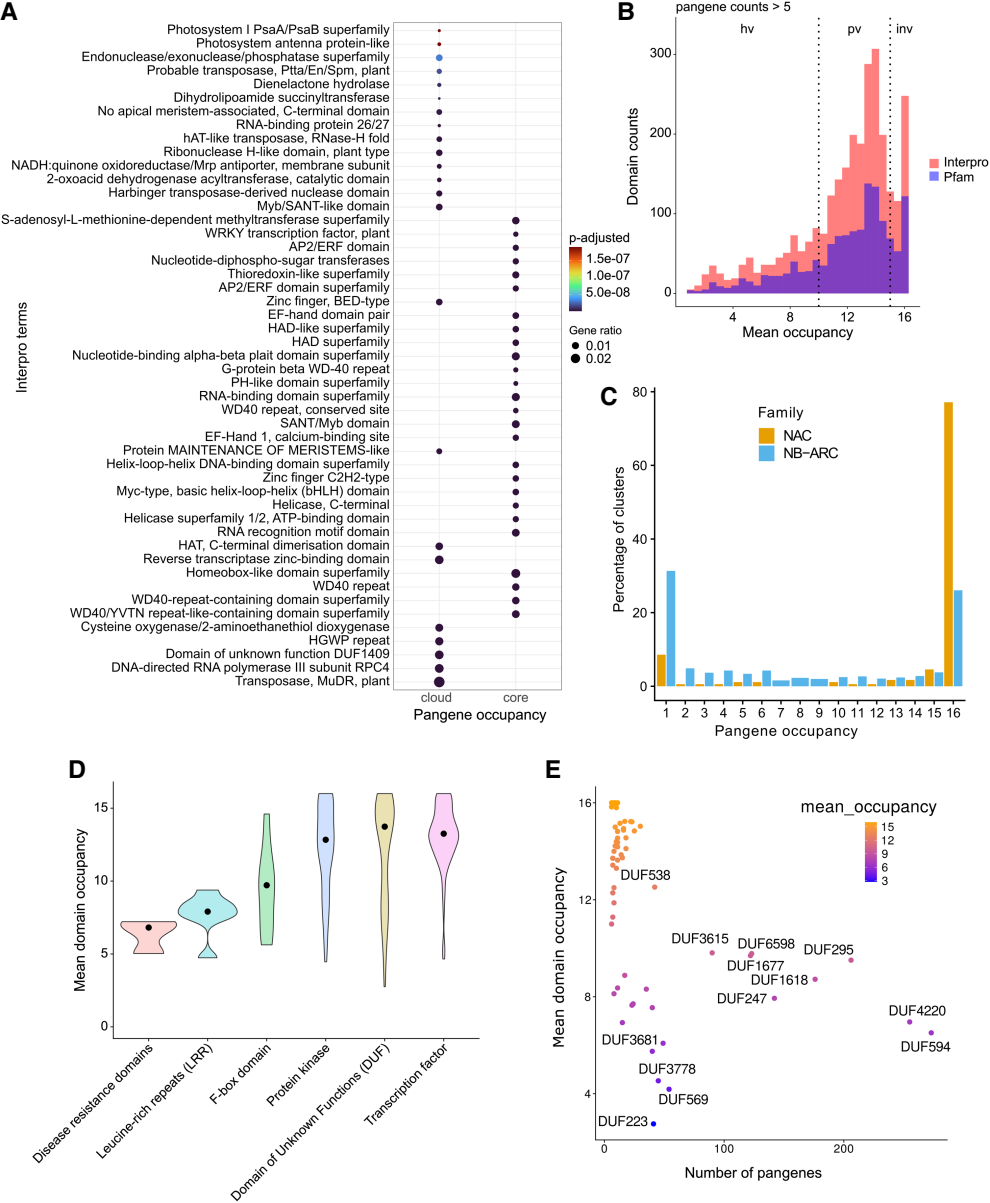
Exploration of predicted pangene domains. (*A*) Enriched InterPro terms among cloud versus core pangenes. The top 30 terms are shown in the plot. Size of the dot represents the enrichment factor, and the color of the dot shows the *P*-value. (*B*) Histogram shows the counts of domains within the pangenes versus their mean domain occupancy for both the Pfam and InterPro domains. The domains were filtered for presence in a minimum of five pangenes. (*C*) The plot shows the percentage of pangene clusters according to their occupancy status for two gene families: NAC domain-containing TFs and NB-ARC domain-containing genes. (*D*) The variation in mean domain occupancy of various Pfam domains for the selected major gene groups is shown. The solid back dot shows the median value. (*E*) The scatterplot shows the mean domain occupancy of various domains under the group “domains with unknown function” (DUFs) versus the number of pangenes with the respective domain. DUFs present in more than 50 pangenes are highlighted in black text.

We identified the most enriched domains found in the set of core versus cloud pangenes and observed some distinct patterns of domain types (Fig. 4A). Core pangenes are enriched in domains including *S*-adenosyl-L-methionine-dependent (SAM) methyltransferase, AP2/ERF, WD40, helix–loop–helix, Myb domain, C2H2 zinc finger, and homeobox domain. Although SAM methyltransferases are responsible for methylation of several substrates, other domains among these pangenes are commonly present in DNA-binding transcription factor (TF) proteins. This enrichment among core pangenes thus reflects the conserved nature of these molecular processes. However, the domains within the cloud pangenes appear to be more varied with transposases seen as a prominent group. The enrichment among cloud pangenes may be indicative of genes that have either a nonconserved nature, or they failed to cluster for a variety of technical reasons.

Among the significantly enriched domains in the cloud pangenes, we found a domain named DNA-directed RNA polymerase III subunit RPC4 (PF05132/IPR007811) that is involved in the transcription of small RNAs (Fig. 4A). There are only four RPC4 pangenes with occupancy = 16, a varying number of shell pangenes, and 228 cloud RPC4 pangenes with occupancy = 1. In Supplemental Figure S4, we show the expansion of this gene family in *indica*- and *aus*-type rice varieties with 15–38 pangenes, whereas *japonica*/*aromatic* rice varieties have only two to three pangenes of this domain, potentially indicating a very substantial expansion of this family in *indica*- and *aus*-type varieties.

Next, we explored the occupancy of protein domains across the pangene set. Variance in the distribution of domains can potentially inform on functional units that either have a tendency to become duplicated or have been more frequently introduced via introgression, compared with those that are invariable. Figure 4B contains a histogram of the mean occupancy of protein domains (InterPro and Pfam) showing a multimodal distribution. As noted in the Methods, we then split the distribution into three groups: “invariable (occupancy) domains,” “partially variable domains,” and “highly variable domains.” We hypothesize that gaining (or losing) copies of invariable domains is highly detrimental to rice. GO enrichment analysis of pangenes containing these groupings of domains provides support for this hypothesis (Supplemental Fig. S5). For example, pangenes containing ubiquitin-protein transferase and TF DNA binding activity show conserved and invariable domains, whereas domains involved in cell surface receptor signaling appear highly variable, indicating the fast-evolving nature of these domains driven by selective breeding pressure, like disease resistance.

As a case study to test the pangene clusters, we analyzed two gene families (Fig. 4C): the NAC TF family (an invariable domain) and the nucleotide-binding domain shared by Apaf-1, certain R-proteins, and CED-4 (NB-ARC) disease-resistance family (a highly variable domain) (Jacquemin et al. 2014). NAC TF family genes have been characterized for their roles in abiotic stress responses in rice (Nakashima et al. 2012). The NB-ARC domain is a conserved nucleotide-binding domain found in a variety of proteins across different organisms, including plants, animals, and fungi. It plays a crucial role in processes like apoptosis (programmed cell death) and innate immune responses. We identified 175 NAC pangenes with 142–154 genes in each genome, and the domain has a mean occupancy of 13.2. 77% of NAC pangenes (n = 135) have core occupancy and 9% cloud (n = 16), indicating a highly invariable domain (Fig. 4C; Supplemental Fig. S6). In comparison, the NB-ARC is a highly variable domain (mean occupancy, 7.2) identifiable within 1005 pangenes with 432–518 genes per genome. NB-ARC family genes are mostly found with an occupancy of 16 or one, indicative of rapid introduction of additional copies or recent gene duplications in some genomes (Fig. 4C; Supplemental Fig. S7). There is also a difference between NAC and NB-ARC in shell pangenes: 9% of NAC pangenes (n = 16) versus 34% of NB-ARC genes (n = 341). One could hypothesize that because NB-ARCs have a role in pathogen effector recognition, it is desirable for the plant to have a wide diversity of genes but to tolerate the loss of a small number of genes in some genomes (leading to shell pangenes), whereas loss of a TF is highly detrimental, and the existence of cloud genes is explained by rare duplication events.

A similar trend is seen with other domains like leucine-rich repeats (LRR; median occupancy, 7.50 ± 0.945), indicating the highly variable nature of these domains (Fig. 4D). Given the role of LRRs in plant immune responses, the domain occupancy data are suggestive that these genes are fast evolving through breeding, for example, when past gene duplications giving resistance to a given pathogen in cultivated rice have been selected. Notably, it is one of the domains associated with pangenes enriched with the highly variable GO term “cell surface receptor signaling pathway” (Supplemental Fig. S5). Multiple domain types with the name “F-box” show a partially variable occupancy (9.87 ± 2.66) compared with the least variable domains of TF (13.3 ± 1.73) or protein kinase (13.4 ± 3.70) types. F-box domain-containing proteins include those involved in the SCF type ubiquitin E3 ligase family and, in plants, have multiple functional roles, including stress responses. Our data also suggest past selective breeding pressure, giving rise to variability in the domain occupancy across the rice pangenome.

The class of “domains with unknown function” (DUFs) shows a median occupancy of 13.6 but with a wider interquartile range (±5.49), with 46 domains present in more than 10 pangenes (Fig. 4E). Several DUFs (DUF594, DUF4220, DUF295, DUF1618, DUF6598, and DUF1668) are present in more than 100 pangenes and have been classified as highly variable domains, which we believe may be indicative of past selection. The genes containing these domains are thus candidates for functional validation and are potential carriers of desirable traits.

### Infrastructure for pangene exploration

**Figure 5.**
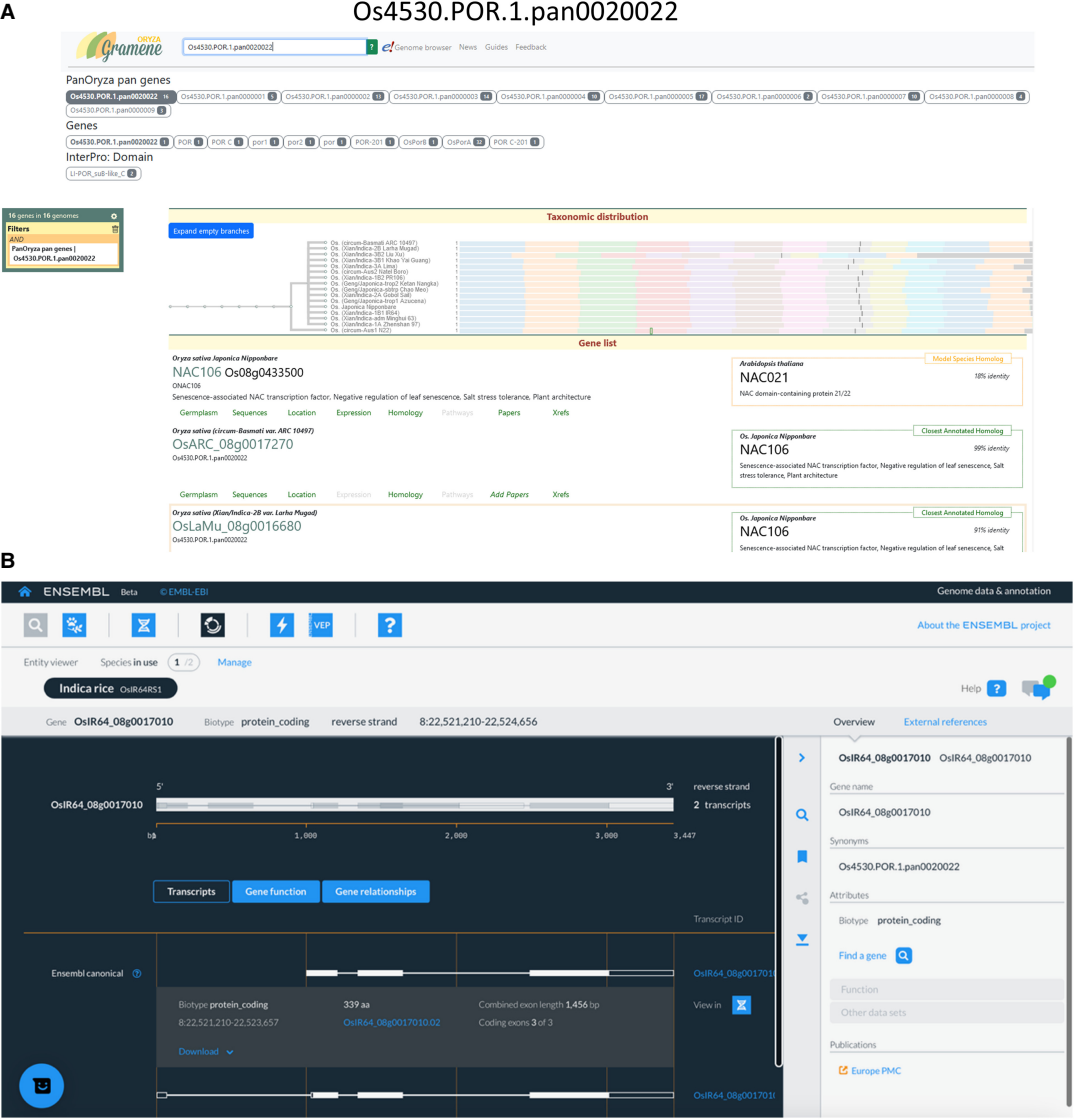
Representative example of infrastructure for pangene exploration using pangene Os4530.POR.1.pan0020022. (*A*) The screen shot shows the record of pangene Os4530.POR.1.pan0020022 on the Gramene database (https://oryza.gramene.org/). (*B*) The page shows the gene record for a member gene OsARC_08g0017270 of Os4530.POR.1.pan0020022 on the Ensembl beta release.

The pangene set has been loaded into popular, widely used databases, including Ensembl Plants and Gramene, and into our own pangene resource (https://panoryza.org/). At Gramene (https://oryza.gramene.org/), it is possible to search with pangene identifiers, for example, Os4530.POR.1.pan0020022, which is the pangene identifier for NAC106 (RAP-DB: Os08g0433500) (Fig. 5A). NAC106 is a NAC TF, which has been associated with salt tolerance, tiller angle, and leaf senescence (Sakuraba et al. 2015). The Gramene search then returns the syntenic and orthologous gene records within each of the genomes assigned to that pangene identifier. There are multiple views for exploring the data, including as phylogenetic trees within the set or as expansions include orthologs from other plants. The RPRP assemblies and annotations have been added into Ensembl Plants, and the new Ensembl site which is currently in beta (beta.ensembl.org). The pangene identifiers are visible as gene synonyms on beta and will become available via Ensembl Plants in a future release (Fig. 5B).

At the PanOryza site, we have a JBrowse (Buels et al. 2016) instance in which each genome can be explored with various tracks of data (Supplemental Fig. S8A). For the IRGSP reference, we have aligned gene models from other cultivars using LiftOff, enabling any variation in models from other genomes to be displayed. For NAC106 as an example, which has occupancy = 16, some variation is suggested within exon 2 in the N22, IR64, and Azucena varieties. Another example is shown in Supplemental Figure S9 that shows the Gramene search result and PanOryza genome browser (JBrowse) for Os4530.POR.1.pan0035634. The members of this pangene coding for NB-ARC domain-containing proteins are present in all *japonica* and Basmati accessions of RPRP but absent in *aus* or *indica* genomes.

We have also created an R library, with code for interactive exploration of clusters using R Shiny through a web browser. The application allows users to visualize the relative position of genes within a cluster across the 16 genomes and explore the members and metadata about each pangene (Supplemental Fig. S8B; https://github.com/PGB-LIV/PanOryza-pan-genes-release-v1.0/tree/main/heatmap_app/).

A permanent DOI has also been created for the pangene matrix and identifiers (https://zenodo.org/records/14772953) so that they can be used now in other research projects. It is anticipated that they will be updated in the future (e.g., yearly), under some of the following circumstances: (1) gene models change; (2) additional genomes are added; and (3) there are refinements to the algorithm for creating pangenes. Full guidelines for the mechanism of updating pangene identifiers are also available at the Ensembl Plants GitHub repository (https://github.com/Ensembl/plant-scripts/tree/master/pangenes). Mappings will be made available so that any users can track how and why pangenes have changed. These resources can all be used by the rice research and breeding community now to assist in mining the wide pool of genetic resources available for Asian rice.

## Discussion

New genomes are frequently being sequenced, assembled, and annotated for many species, although structural genome annotation remains very challenging to perform accurately (Salzberg 2019). Archival repositories like INSDC contain an ever-increasing number of genome assemblies and annotations of variable quality, and it is highly challenging for most research groups to interpret. For example, asking how a given gene varies in presence, absence, or expression across different rice varieties could not previously be performed without running complex, custom-built bioinformatics analyses. Our pangene set allows for identification of alleles across varieties, as well as cases in which protein sequences differ across varieties from the same chromosomal sequence, indicative of functional diversification, pseudogenization, or gene model inaccuracies, which can be explored.

Other groups have also explored or generated pangenomes for rice. For example, Shang et al. (2022) sequenced, assembled, and annotated a 251 accession panel, demonstrating, as in our work, that LRR genes have variable copy number across genomes. Wu et al. (2023) generated a pangenome from 74 weedy and cultivated rice accessions, building ortholog relationships using BLASTP, shedding light on genes involved in domestication. However, no direct resources were made available to explore these pangenomes and straightforwardly identify orthologs/syntelogs in either of the above-mentioned studies. Our work has a different focus in that we have created a robust and stable pangene set for Asian rice based on WGA of 16 very high-quality assemblies and gene annotation sets, supported by multiomic experimental data. WGA gives better calling of orthologs versus paralogs compared with a BLASTP-based approach (as illustrated in examples below) and allows for the identification when protein sequences differ greatly from near identical chromosomal sequence (for examples, see Supplemental Fig. S10).

The Rice Gene Index (RGI; https://riceome.hzau.edu.cn) resource (Yu et al. 2023) has also created ortholog groups across the RPRP, based on reciprocal best match method, creating more than 110,000 groups via this method. After filtering gene models from different input sets, our 78,000 pangenes have reasonable agreement, with about 22,000 pangenes having identical membership to RGI clusters (Supplemental Fig. S11A). As demonstrated in Supplemental Figure S7, the NB-ARC gene family has rapidly expanded with many gene duplications leading to pangenes with low occupancy. In Supplemental Figure S11C, we compare the clusters formed for NB-ARC between PanOryza (this work) with RGI. The RGI algorithm forms clusters based on protein sequence similarity (instead of syntenic orthologs from genome alignments), causing many NB-ARC sequences to be assigned to the same cluster (Supplemental Fig. S11C). The RGI data for NB-ARCs has many large clusters with 25 to more than 200 members, indicating that paralogs have been clustered together, which is less useful for finding novel genes with newly adapted functions. In Supplemental Figure S11B, we can also see the NAC genes are also more tightly clustered into core families (occupancy close to 16) in PanOryza compared with RGI, which gives a much wider distribution of membership within clusters, indicating less good resolution of orthologs and paralogs.

We find a reasonable agreement of pangene membership identified using GET_PANGENES or with GENESPACE (Lovell et al. 2022), which also generates syntenic orthogroups using a BLASTP-like approach on protein sequences (Supplemental Figs. S12, S13). Nearly 40,000 PanOryza pangenes show >70% similarity with those generated using GENESPACE and about 27,000 pangenes with identical membership. The median size of clusters is also comparable for the two gene families NAC (GENESPACE:16; PanOryza:18) and NB-ARC (GENESPACE:10; PanOryza:8) (Supplemental Fig. S13). However, we see that GENESPACE has apparently less resolution of orthologs and paralogs within the NAC family of TFs, owing to the underlying protein similarity algorithm.

A recent study (Guo et al. 2025) also explored wild and cultivated rice accessions to construct a pangenome, however again based on the CDS alignment, and then used it to classify genes based on their frequency into different categories, which will not adequately differentiate orthologs and paralogs for complex gene families. Thus, we conclude that future studies on an expanded panel of rice accessions must rely on high-quality genome assembly with consistent annotation and a WGA approach for identification of pangenes.

In our study, persistent identifiers have been assigned to pangenes, along with a system allowing for pangenes to be updated over time as source data or evidence changes, with the ultimate aim of making these resource straightforwardly available in end-user focused databases. Therefore, these resources have been made openly available through Ensembl Plants and Gramene for further exploration and identification of variations within a pangene as well as for the presence or absence of genes in certain rice accessions. The availability of pangenes in popular public databases allows the rice research community to move now to working in the pangenome context. For example, groups planning new genome-wide association, gene expression, or proteomics studies can select to use the reference assembly (and gene/protein set) most closely matching their variety of interest but can use the pangene set created here to compare easily when SNPs/genes/proteins are present or absent across rice families, including in the reference genome IRGSP.

### Interpretation of pangenes by their occupancy across genomes

Our QC metrics demonstrated that clusters are robustly formed, for example, containing gene/protein sequences that are highly related across genomes but with some outliers in which sequences have low similarity. The clustering algorithm ensures that overlapping genes on different strands are not placed in the same cluster, but there are multiple other reasons why clusters contain only moderately similar or unrelated protein sequences. These include cases in which one coding sequence has been predicted within introns, within UTRs, or on a different frame from another gene model. In a few cases, the differences could be the result of true biological differences, but perhaps a default assumption should be that for incongruous pangenes, some gene models are incorrect in one or more cultivars. The metrics we have generated allow such clusters to be identified, and work can begin to identify the correct coding sequence.

Exploring occupancy data points to several key conclusions. First, core pangenes are well supported by experimental evidence (transcriptomics, proteomics) and have easily recognizable protein domains and structures. As a general trend, the extent and quality of experimental evidence rapidly decreases with occupancy: In some cases, there are likely pseudogenes or only partial gene models that have been incorrectly predicted. In other cases, as demonstrated in the exploration of mean domain occupancy, genes under selective pressure have more variable occupancy.

### Implications for rice research and breeding

The concept of a reference genome remains a useful one, despite the wealth of genomes now available, as it is a very costly and challenging exercise to annotate a genome to a very high quality. However, it is evident that the IRGSP RefSeq does not fully represent the coding potential of the diverse set of varieties being grown around the world. In this pangene set, we find more than 5000 genes that are observed in at least two other *Oryza* subpopulations but absent in IRGSP. Through specific examples, we have shown how reference-free clustering can help in identification of pangenes absent in a reference genome like Nipponbare, a *japonica* accession, or those pangenes that are absent in a subpopulation like *indica* or *japonica*. Alternatively, NB-ARC pangene like Os4530.POR.1.pan0035634 present only in *japonica* and Basmati makes a case study for disease-resistance candidate pangenes.

Another key finding of this study is that by exploring the pangene set, we can see clear signatures of selection within protein domains. For example, some highly variable domains are associated with immune-related functions and are presumably resistant to pathogens and pests. The expansion of RNA polymerase III subunit C4 (RPC4) domain-containing proteins in the *aus* and *indica* accessions is an intriguing finding of the study that needs further validation. One of the Nipponbare member of RPC4 domain pangene Os4530.POR.1.pan0011131, which has been previously characterized (Os04g0394500/ *DGS1*), is associated with pollen sterility in an interspecific hybrid, and gene duplication is proposed as a key factor for its variable number within the *Oryza* sp. (Nguyen et al. 2017). At this point, it can only be speculated if the varying number of RPC4 genes might have led to reproductive isolation barriers among rice subpopulations. Through identification of genes carrying currently understudied domains (DUFs) and of genes with recognizable domains that are present in only some subpopulations, these resources are ready for mining to find new alleles with desirable traits. Lastly, our approach for creating pangenes and assigning globally unique (but easily updatable) stable identifiers can be adopted by other communities attempting to deal with the complexity of multiple genomes and annotation sets within a species of interest.

## Methods

### Consistent gene annotation for the RPRP

To create the assembly of pangene sets across *O. sativa*, we first collected whole-genome sequences, gene and protein sequences (all FASTA format), and gene model coordinates (GFF format) for the RPRP rice population. We used as input to pangene set building gene models generated by a single consistent pipeline, as described previously (Zhou et al. 2023) for all 16 genomes. In brief, models were generated with MAKER-P, with ab initio gene prediction (SNAP [Korf 2004], AUGUSTUS [Stanke et al. 2008], and FGENESH [Solovyev et al. 2006]), deep RNA-seq data (long and short read), and assignment of canonical gene models using TRaCE (Olson and Ware 2021). For the IRGSP (Nipponbare) reference, we created a merged annotation set from the three sources of gene models (RAP-DB; a new model following the pipeline above, called OsNip; and MSU), using the algorithm below. For practical purposes, the merged set allows a researcher to find a given RAP-DB or MSU identifier in the pangene set, while giving an opportunity to compare statistical attributes of genes/proteins by comparing the OsNip annotation with the other 15 (see as an example Nipponbare gene Os01g0103600 in Supplemental Fig. S14).

Protein-coding genes from the three sets were merged based on gene overlap calculated using chromosomal coordinates and strand information. Genes were merged (protein-coding genes with CDS transcripts only) if the gene ranges overlapped by ≥50% of the length of the shorter gene. The merged gene's coordinates were adjusted to match the most extreme 5′ and 3′ coordinates of any protein-coding transcript within the merged gene. The name given to the merged gene was assigned from one of its members, giving priority to the RAP-DB identifier, followed by the OsNip identifier, and finally the MSU identifier. The MSU annotation set uniquely contain some genes tagged as transposable elements (TEs), which were not filtered at source (in case they had been incorrectly annotated as TEs) but for some analyses were later removed, as detailed below.

### Building pangene clusters and quality control

We ran the GET_PANGENES pipeline (version 11012024) (Contreras-Moreira et al. 2023), which performs WGA using minimap2 (version 2.17) (Li 2018), followed by BEDTools intersect (Quinlan and Hall 2010) to determine overlaps in each pairwise alignment. All haploid gene models progressed to cluster building, except for the Liu Xu annotation set, in which alternative heterozygous alleles of genes, which had been placed onto contigs as part of a diploid genome resolution process, were excluded from the pangene clustering step. Genome sequences and GFF3 files for the RPRP were sourced from Ensembl Plants (release 57; unchanged in current release 60) and OsNip from Gramene.

GET_PANGENES was used to generate quality control metrics, including running BUSCO (v 5.7.1); Clustal Omega (version 1.2.4) (Sievers et al. 2011) was used to generate protein-level multiple sequence alignments for each pangene cluster; and AliStat (version 1.14) (Wong et al. 2020) was used to generate distance metrics. Core gene sets counts were generated using Tettelin (Tettelin et al. 2005) and Willenbrock (Willenbrock et al. 2007) functions, fitted after 20 permutation experiments.

Pangenes were classified as *core* if they contained a member from 100% of input genomes (16 in this case); *softcore*, 95% of genomes (15); *cloud*, one or two genomes; and *shell*, (all other cases) (Contreras-Moreira et al. 2022). AED scores were generated by TRaCE, using RNA-seq data sourced from Zhou et al. (2023). Each transcript was assigned an AED score, reflecting the proportion of the transcript overlapped by a StringTie model from each of the three RNA-seq libraries.

Paralog and ortholog counts were generated from Ensembl Compara (build 111) (for details, see Supplemental Methods; https://ftp.ebi.ac.uk/ensemblgenomes/pub/plants/release-58/tsv/ensembl-compara/homologies).

AlphaFold models for *O. sativa* (reference id: UP000059680) were retrieved from the AlphaFold protein structure database (https://alphafold.ebi.ac.uk/) and the mean prediction score calculated for each model. Gene expression data for 11,726 rice RNA-seq samples were sourced from https://plantrnadb.com. Average FPKM values were calculated for each RAP-DB/MSU gene and assigned to the precomputed pangene cluster.

### Assigning stable identifiers

We have designed an approach for assigning stable identifiers to pangenes (following similar proposals made by https://www.agbiodata.org/), which may be repurposed for other plant genomes, as follows: [clade].[group].[version].panddddddd, where clade is a two-letter code for a species-level pangene or a one-letter code for the genus level, followed by the NCBI taxon ID, such as Os4530 for *O. sativa*; group is a unique three-letter code for the consortium/group releasing the pangene set, for example, POR for our “PanOryza” consortium; version is integers starting from one, incremented for each new release; and panddddddd is the pangene identifier, with digits 0000001 for pangene clusters with two or more members and 1000001 for singleton clusters (containing only one gene).

The identifiers thus have the property that they can be globally unique but can be produced by different consortia, using different sets of input genomes. We assigned pangene identifiers to all genes clustered as part of this pangene set. New releases could be created in the following circumstances: changes to genome assemblies or gene models, addition of new genomes or gene models, and changes to the clustering algorithm.

### Generating a rice panproteome map

We next determined the experimental support for proteins in the rice panproteome, by performing large-scale reanalysis of public proteomics data. Data sets were sourced from the ProteomeXchange central repository (Vizcaino et al. 2014), using the inclusion criteria (on data sets released up to the May 2023) that a global (nonenriched) analysis had been performed on any rice tissue, sourced from any *O. sativa* variety, using a Thermo Fisher Scientific instrument (for simplicity of pipeline compatibility), and the source article mentioned more than 1000 proteins identified. For further details, see Supplemental Methods. The results of this process are available at https://peptideatlas.org/builds/rice/.

### Assignment of protein domains to pangenes

Domains in the protein annotations of all 18 annotation sets (for 16 genomes) were identified using InterproScan-v5.62-94.0 and mapped onto pangenes and source gene models. We next assigned a representative domain for the cluster as the most common protein domain found across genomes (in the case of more than one domain being tied as “most common,” one is randomly selected). Using this most common protein domain (for both InterPro and Pfam), the consistency of the pangene clusters was checked to identify the genomes in which the representative domain is present. Next, for every InterPro and Pfam domain, the “domain occupancy” was first calculated for each pangene across 16 genomes, namely, counting the number of genomes with a protein carrying the domain within the pangene (for IRGSP, only OsNip assignments were included for this analysis to avoid biasing the Nipponbare domain counts). Next, the “mean domain occupancy” was calculated as the mean of the domain occupancy across all pangenes in which the domain can be found. Domains were filtered to include only those found in five or more pangenes and were categorized based on mean occupancy across pangenes as “highly variable” when mean occupancy was less than 10; “partially variable,” more than or equal to 10 and less than 15; and “invariable,” equal or more than 15. Interpro2Go annotations were used for identification of GO terms. Top terms enriched within these pangenes were identified using clusterProfiler (v4.6.2) (Wu et al. 2021) with a *P*-value cutoff of < 0.05, adjusted by the Benjamini–Hochberg method. To identify the most significant InterPro domains in core versus cloud pangenes, we first filtered out clusters containing only TEs. Because regular protein-coding genes could be among the clusters that were labeled as TEs in past Nipponbare annotations (sourced from MSU), for pangenes with occupancy of one, we removed the cluster if it is annotated as a TE in the InterProScan results or the original MSU annotations. Further, for pangenes with occupancy greater than one, a cluster was filtered out only if all the genes in a cluster are identified as TEs in the InterProScan results. With these pangenes filtered for TEs, we used the enricher function of the clusterProfiler as above to identify the significant InterPro term in each occupancy class (*P*-value = 0.05, adjusted by Benjamini–Hochberg method). The redundant term descriptions within each occupancy class were collapsed using the stringdist function (method, Jaro–Winkler distance). The top 30 terms for the core and cloud occupancy class based on the adjusted *P*-value were used for the dotplot, in which the size and color of the dot show the GeneRatio (term enrichment factor) and adjusted *P*-value, respectively.

The NAC TF gene family and NB-ARC domain-containing proteins were identified and analyzed for their genome occupancy as described in the Supplemental Methods.

### Infrastructure development

The PanOryza site incorporated JBrowse (version 1.16.11). For display purposes, gene models from other genomes were mapped to the Nipponbare reference using LiftOff (v1.6.3) (Shumate and Salzberg 2021). The interactive heatmap visualization of pangenes was created using R package InteractiveComplexHeatmap (Gu et al. 2022). As noted in the results, pangenes identifiers have been loaded into Ensembl Plants and Gramene for querying. The protein sets derived from the RPRP set are also available within the UniProt Knowledge base (The UniProt Consortium 2025; https://www.uniprot.org/proteomes?query=oryza+sativa).

### Comparison of pangenes with other pipelines

We compared pangenes from this work with the “Ortholog Gene Indices” (OGI clusters) from the RGI (https://riceome.hzau.edu.cn) and with those generated using GENESPACE (Lovell et al. 2022). The percentage of similarity was calculated between the matched clusters based on agreement of identifiers in each pangene (Supplemental Methods).

### Code availability

Code is available at GitHub (GET_PANGENES: https://github.com/Ensembl/plant-scripts/blob/master/pangenes/; Nipponbare merged genes: https://github.com/Ensembl/plant-scripts/tree/master/scripts). The code for recreating analyses in this paper is also available at GitHub (https://github.com/PGB-LIV/PanOryza-pan-genes-release-v1.0) and as Supplemental Code.

## Data access

The input data for running GET_PANGENES pipeline and the output files generated by the pipeline have been deposited at Zenodo (https://zenodo.org/records/14772953).

## Supplemental Material

Supplement 1

Supplement 2

Supplement 3

Supplement 4

Supplement 5

Supplement 6

Supplement 7

Supplement 8

Supplement 9

Supplement 10

Supplement 11
