# Supplementary material for: The PanOryza pangene catalog of Asian cultivated rice
Source: Genome Res. 2026 Jan;36(1):226–38. doi: 10.1101/gr.280790.125 (PMC12758395; doi:10.1101/gr.280790.125)
Supplement: Supplement 1 [file Supplemental_methods.pdf]

Supplemental methods

Generating a rice pan-proteome map

Raw MS data were converted to mzML format (Martens et al. 2011), using ThermoRawFileParser (Hulstaert et al. 2020). Datasets were searched with the parameters listed in Supplementary table S7, using the MSFragger search engine (Kong et al. 2017) (version 3.5), against a protein database formed by merging the 16 protein sets from the RPRP gene models, as well as the RAP-DB (canonical and predicted proteins, version 2022-03-11) and MSU (version 7) protein sets. Common contaminants were added from the cRAP database (https://www.thegpm.org/crap/), and reversed decoy sequences added using the FragPipe utility. Peptide-spectrum matches from MSFragger in PepXML format (Keller et al. 2005) were post-processed with the Trans-Proteomic Pipeline version 6 (Deutsch et al. 2023) (PeptideProphet (Keller et al. 2002), iProphet (Shteynberg et al. 2011), ProteinProphet (Nesvizhskii et al. 2003)). The 19 datasets that passed quality control (>500 proteins identified) were loaded into PeptideAtlas after thresholding the identifications such that the resulting protein-level FDR was 1%.

Paralog and ortholog counts generation in Ensembl Compara

To generate this dataset, representative protein sequences of coding genes, from 116 plant genomes, were classified into clusters against a profile HMM library incorporating resources such as PANTHER (Thomas et al. 2022) and TreeFam (Ruan et al. 2008); with any remaining unclassified genes being included in a process of clustering by hcluster_sg (https://sourceforge.net/p/treesoft/code/HEAD/tree/trunk/hcluster/) using the results of a BLAST search (Camacho et al. 2009). Each cluster's protein sequences were aligned by MCoffee (Wallace et al. 2006) or MAFFT (Katoh and Standley 2013), and for the resulting alignments, TreeBeST (TreeFam) was used to generate a gene tree in which nodes were annotated as representing events such as a gene duplication or a speciation. Homology relationships were inferred from the annotated gene tree, with the type of homology between a given gene pair being determined by features of their evolutionary history, such as whether their last common ancestor represented a speciation or duplication event (Vilella et al. 2009; Herrero et al. 2016). Source data is also available at https://plants.ensembl.org/info/genome/compara/index.html.

Analysis of gene families

For the NAC transcription factor (TF) gene family, query protein sequences from Plant TFDB (Jin et al. 2017) were used for BLASTP blastP (ncbi-blast, v2.12.0, e-value < 1E-05) and HMMER (HMMER 3.3, e-value < 1E-3) search against protein sequences from all gene models within the pan-genes. Results were checked for Pfam (PF02365) and InterPro domain IPR003441, IPR044799 or IPR036093, and

pan-genes containing at least one protein with the domain were retained and labeled positive for 'NAC'.

The identification of NB-ARC domain-containing proteins in RPRP lines was carried out using the Gramene Oryza API (Tello-Ruiz et al. 2021). A targeted query was employed to retrieve genes annotated with the InterProScan domain IPR002182 (NB-ARC) from gene trees. The query output, provided as a CSV file, was then filtered by the system name field to isolate entries corresponding to RPRP genomes. These filtered genes were subsequently mapped to their respective pan-gene clusters and were designated "NB-ARC" if any pan-gene member from a genome was identified within the NB-ARC domain-containing gene set.

Comparison of pan-genes with other pipelines

For pan-gene clusters generated by GET-PANGENES against those generated by Rice Gene Index (OGI clusters), we first matched clusters that shared the same RAP-DB (Nipponbare) identifier, and excluded Minghui 63, Zhen Shan 97 and Gramene annotations as these were created using different set of identifiers / gene model annotations. For the remaining members, we calculated the percentage agreement of identifiers within each pan-gene. For this, the percentage similarity was calculated as matching counts / total counts where total counts is the number of unique identifiers in a combined set of OGI and pan-genes; matching counts is the number of identifiers matched between OGI and pan-gene.

To compare the pan-gene clusters generated by GET-PANGENES against GENESPACE (Lovell et al. 2022), all the protein sequences in our pan-gene clusters were combined and parsed based on individual genomes. GENESPACE was run as instructed on https://github.com/jtlovell/GENESPACE. Comparison of individual pan-genes generated by the two pipelines were carried out as mentioned above for RGI except that no genomes were excluded for comparison with GENESPACE. The matched clusters were filtered for NAC/NB-ARC pan-genes for plotting based on the size of individual pan-gene clusters.

Supplemental References

Camacho C, Coulouris G, Avagyan V, Ma N, Papadopoulos J, Bealer K, Madden TL. 2009. BLAST+: architecture and applications. *BMC Bioinformatics* **10**: 421.

Deutsch EW, Mendoza L, Shteynberg DD, Hoopmann MR, Sun Z, Eng JK, Moritz RL. 2023. Trans-Proteomic Pipeline: Robust Mass Spectrometry-Based Proteomics Data Analysis Suite. *J Proteome Res* **22**: 615–624.

Herrero J, Muffato M, Beal K, Fitzgerald S, Gordon L, Pignatelli M, Vilella AJ, Searle SMJ, Amode R, Brent S, et al. 2016. Ensembl comparative genomics resources. *Database : the journal of biological databases and curation* **2016**: bav096.

Hulstaert N, Shofstahl J, Sachsenberg T, Walzer M, Barsnes H, Martens L, Perez-Riverol Y. 2020. ThermoRawFileParser: Modular, Scalable, and Cross-Platform RAW File Conversion. *Journal of Proteome Research* **19**: 537–542.

Katoh K, Standley DM. 2013. MAFFT multiple sequence alignment software version 7: improvements in performance and usability. *Mol Biol Evol* **30**: 772–780.

Keller A, Eng J, Zhang N, Li X, Aebersold R. 2005. A uniform proteomics MS/MS analysis platform utilizing open XML file formats. *Mol Syst Biol* **1**.

Keller A, Nesvizhskii AI, Kolker E, Aebersold R. 2002. Empirical Statistical Model To Estimate the Accuracy of Peptide Identifications Made by MS/MS and Database Search. *Anal Chem* **74**: 5383–5392.

Kong AT, Leprevost FV, Avtonomov DM, Mellacheruvu D, Nesvizhskii AI. 2017. MSFragger: ultrafast and comprehensive peptide identification in mass spectrometry-based proteomics. *Nat Methods* **14**: 513–520.

Lovell JT, Sreedasyam A, Schranz ME, Wilson M, Carlson JW, Harkess A, Emms D, Goodstein DM, Schmutz J. 2022. GENESPACE tracks regions of interest and gene copy number variation across multiple genomes. *eLife* **11**: e78526.

Martens L, Chambers M, Sturm M, Kessner D, Levander F, Shofstahl J, Tang WH, Römpp A, Neumann S, Pizarro AD, et al. 2011. mzML—a Community Standard for Mass Spectrometry Data. *Molecular & Cellular Proteomics* **10**: R110.000133.

Nesvizhskii AI, Keller A, Kolker E, Aebersold R. 2003. A Statistical Model for Identifying Proteins by Tandem Mass Spectrometry. *Anal Chem* **75**: 4646–4658.

Ruan J, Li H, Chen Z, Coghlan A, Coin LJM, Guo Y, Hériché J-K, Hu Y, Kristiansen K, Li R, et al. 2008. TreeFam: 2008 Update. *Nucleic Acids Res* **36**: D735-740.

Shteynberg D, Deutsch EW, Lam H, Eng JK, Sun Z, Tasman N, Mendoza L, Moritz RL, Aebersold R, Nesvizhskii AI. 2011. iProphet: multi-level integrative analysis of shotgun proteomic data improves peptide and protein identification rates and error estimates. *Molecular & Cellular Proteomics : MCP* **10**: M111 007690.

Tello-Ruiz MK, Naithani S, Gupta P, Olson A, Wei S, Preece J, Jiao Y, Wang B, Chougule K, Garg P, et al. 2021. Gramene 2021: harnessing the power of comparative genomics and pathways for plant research. *Nucleic Acids Research* **49**: D1452–D1463.

Thomas PD, Ebert D, Muruganujan A, Mushayahama T, Albou L-P, Mi H. 2022. PANTHER: Making genome-scale phylogenetics accessible to all. *Protein Sci* **31**: 8–22.

Vilella AJ, Severin J, Ureta-Vidal A, Heng L, Durbin R, Birney E. 2009. EnsemblCompara GeneTrees: Complete, duplication-aware phylogenetic trees in vertebrates. *Genome Res* **19**: 327–335.

Wallace IM, O'Sullivan O, Higgins DG, Notredame C. 2006. M-Coffee: combining multiple sequence alignment methods with T-Coffee. *Nucleic Acids Res* **34**: 1692–1699.
