# Supplementary material for: The PanOryza pangene catalog of Asian cultivated rice
Source: Genome Res. 2026 Jan;36(1):226–38. doi: 10.1101/gr.280790.125 (PMC12758395; doi:10.1101/gr.280790.125)
Supplement: Supplement 2 [file Supplemental_figures.pdf]

Supplemental Information

PanOryza: A publicly accessible pan-gene catalog of Asian cultivated rice

Bruno Contreras-Moreira[1,3], Eshan Sharma[2,*], Shradha Saraf[3,*], Guy Naamati[3], Parul Gupta[4], Justin Elser[4], Dmytro Chebotarov[5], Kapeel Chougule[6], Zhenyuan Lu[6], Sharon Wei[6], Andrew Olson[6], Ian Tsang[12,13], Disha Lodha[3], Yong Zhou[7], Zhichao Yu[8], Wen Zhao[8], Jianwei Zhang[8], Sandeep Amberkar[2], Kawinnat Sue-Ob[2], Zhi Sun[10],Maria Martin[3], Kenneth L. McNally[5], Doreen Ware[6,9], Eric W Deutsch[10], Dario Copetti[11], Rod A. Wing[7,11], Pankaj Jaiswal[4], Sarah Dyer[3], and Andrew R Jones[2,^]*

Supplemental figures S1-S14

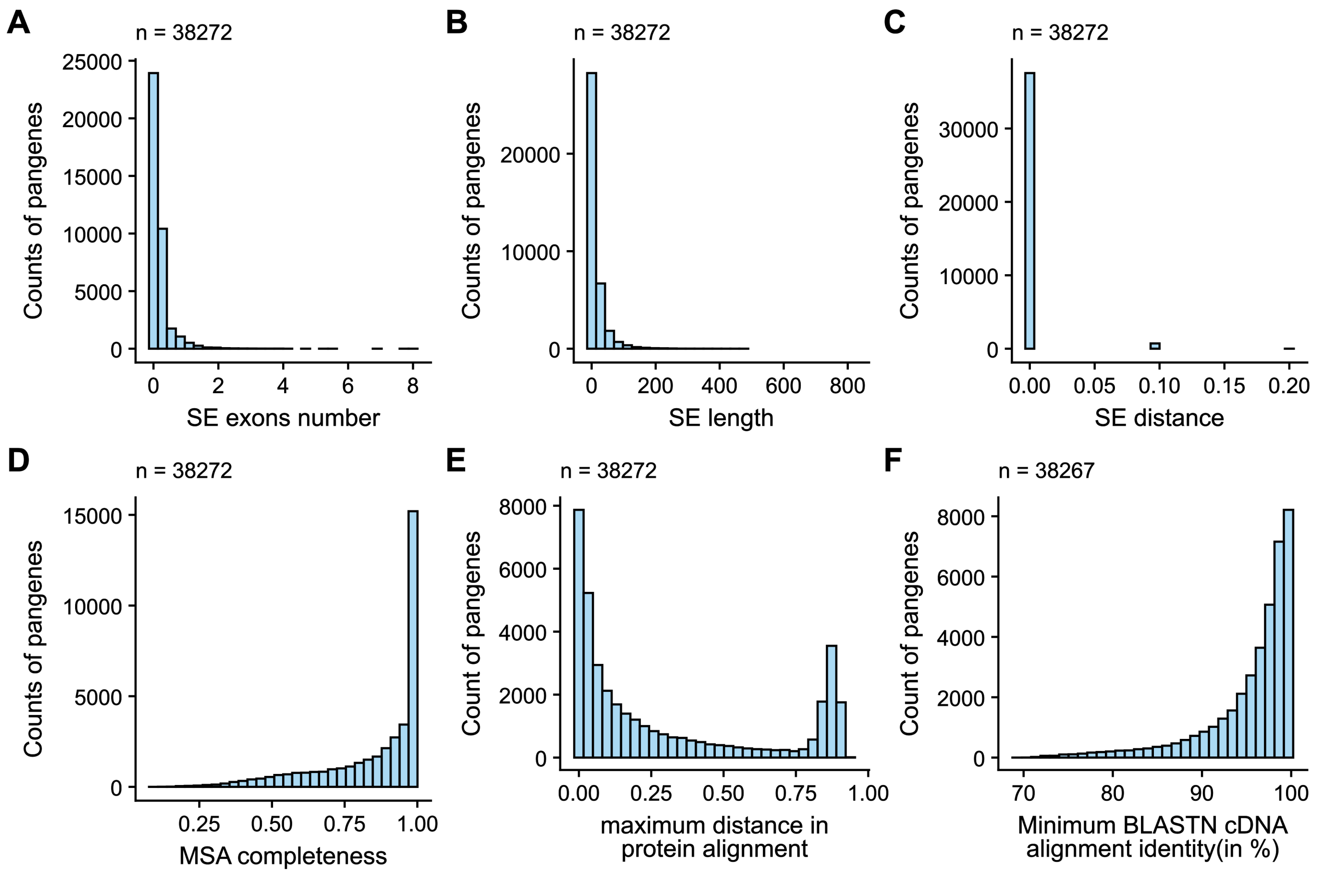

Supplemental figure S1. Quality control statistics of pan-genes. A) The standard error (SE) in the count of exons, B) SE of protein length, C) SE of sequence distance among all pan-gene members (transcripts/proteins) is plotted against the counts of pan-genes. D) The completeness of multiple sequence alignment (Ca values, calculated as in Wong et. al. 2020) and E) maximum sequence distance among pan-gene members is plotted against the counts of pan-genes. F) Minimum BLASTN cDNA alignment identity among members of pan-gene is shown against the number of pan-genes.

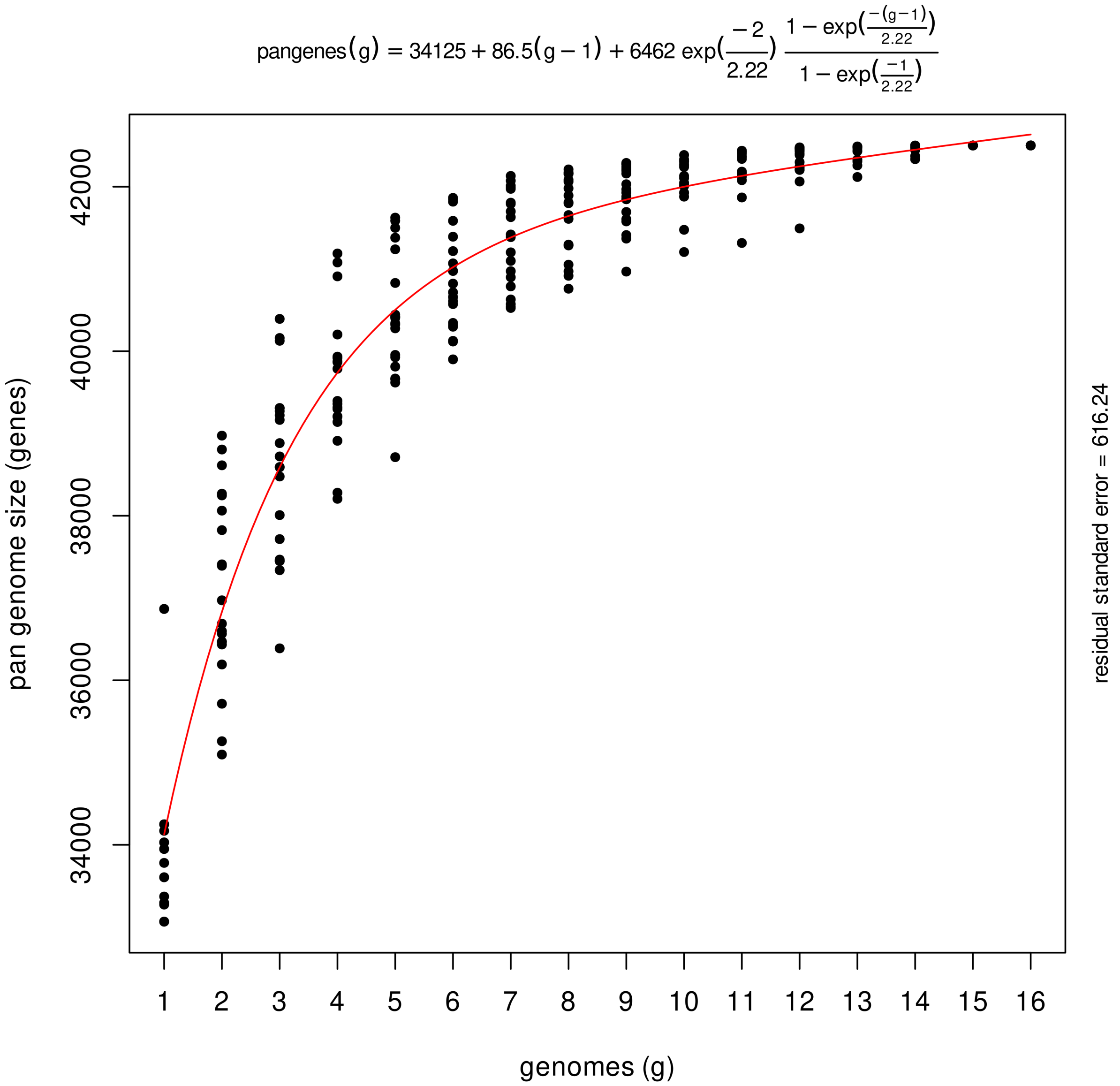

Supplemental figure S2. Pan-genome growth after adding RPRP accessions in random order, yielding 42,501 pan-genes. Tettelin function (red) was fitted after 20 permutation experiments. Pangenes encoded only in one assembly were excluded to reduce potential annotation artifacts.

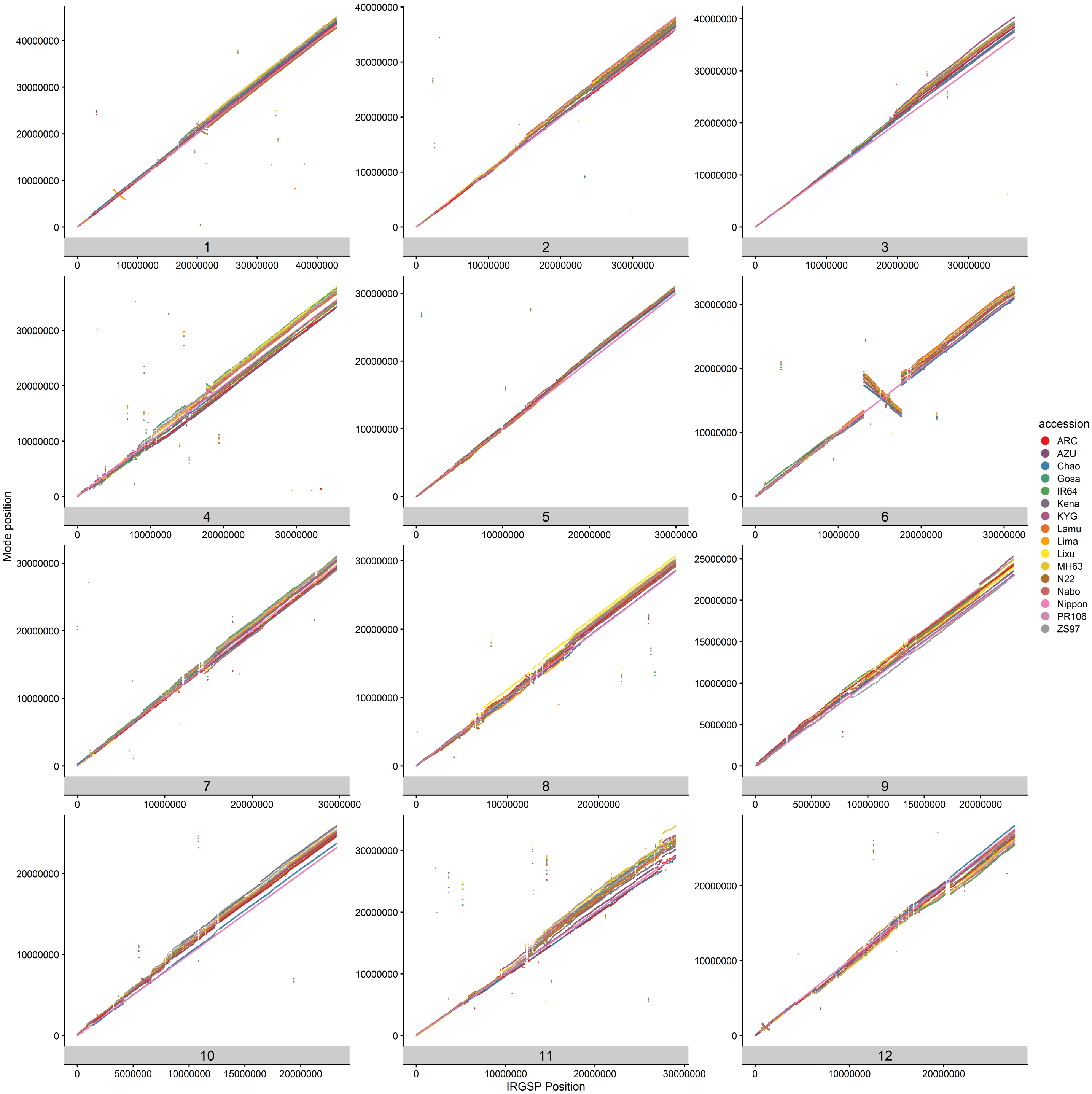

Supplemental figure S3. Position of genes within pan-gene clusters on the chromosomes across the RPRP accessions. Scatter plots show the mode position of genes across the RPRP on each of the 12 rice chromosomes (y-axis) versus the position on the IRGSP reference genome on the x-axis. The names of accessions have been abbreviated as follows – ARC: ARC 10497 , AZU: Azucena , Chao: CHAO MEO , Gosa: GOBOL SAIL (BALAM) , IR64: IR 64 , Kena: KETAN NANGKA , KYG: KHAO YAI GUANG , Lamu: LARHA MUGAD , Lima: LIMA, Lixu: LIU XU , MH63: Minghui 63 , N22: N22 , Nabo: NATEL BORO , Nippon: Nipponbare , PR106: PR 106)

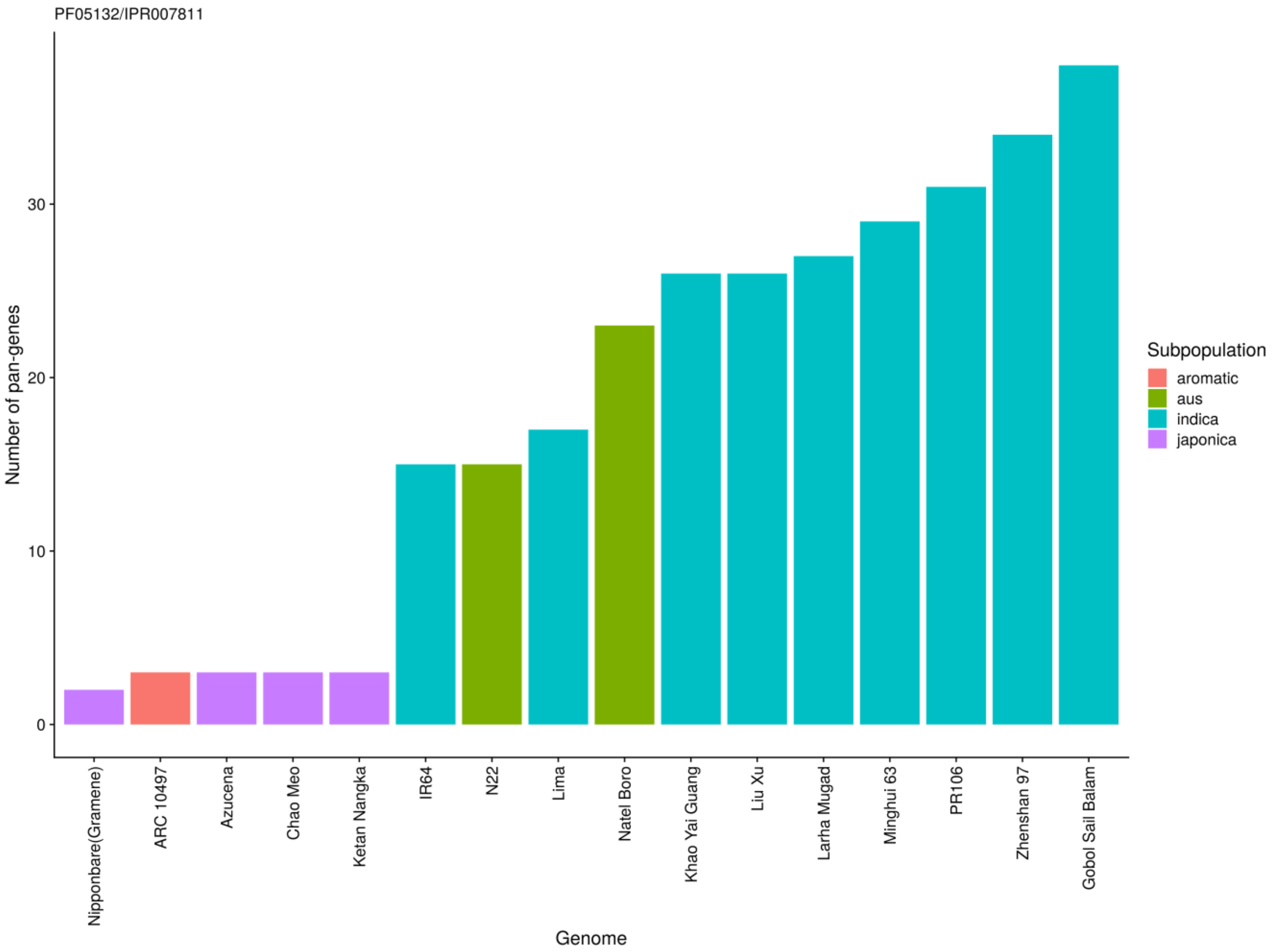

Supplemental figure S4. Expansion of RNA polymerase III subunit C4 (RPC4) domain containing genes in *aus* and *indica*. The bar plot shows the number of pan-genes on the y-axis with their member proteins containing PF05132/IPR007811 domain in various RPRP genomes on X-axis. The bars are colored according to the subpopulation of rice. Note: For consistent comparison, RAP-DB and MSU based Nipponbare annotations were removed for the analyses.

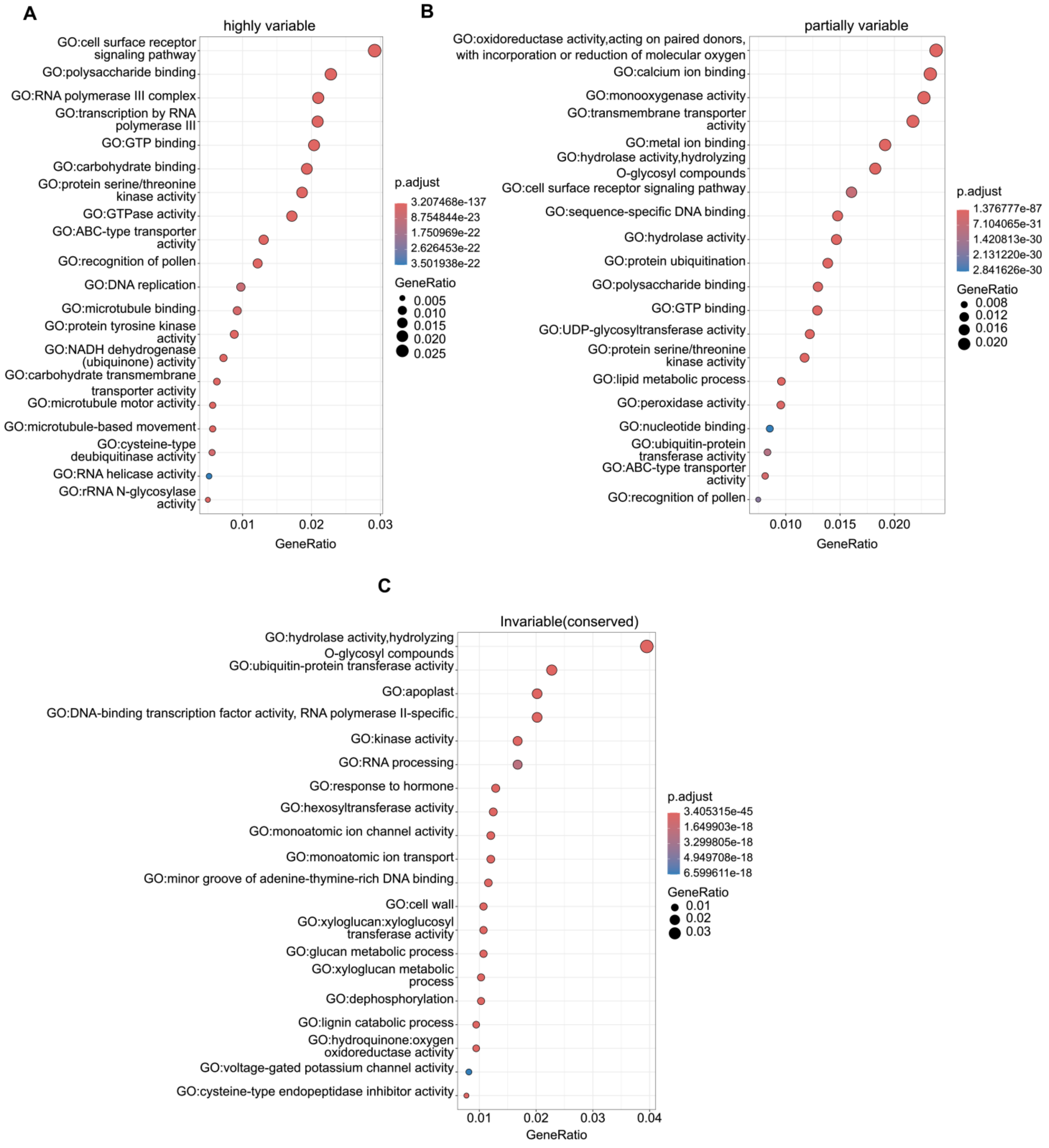

Supplemental figure S5. GO enrichment analysis for domains of variable occupancy. Most significant gene ontology terms for proteins containing domains classified as A) highly variable, B) partially variable or C) invariable (or conserved).

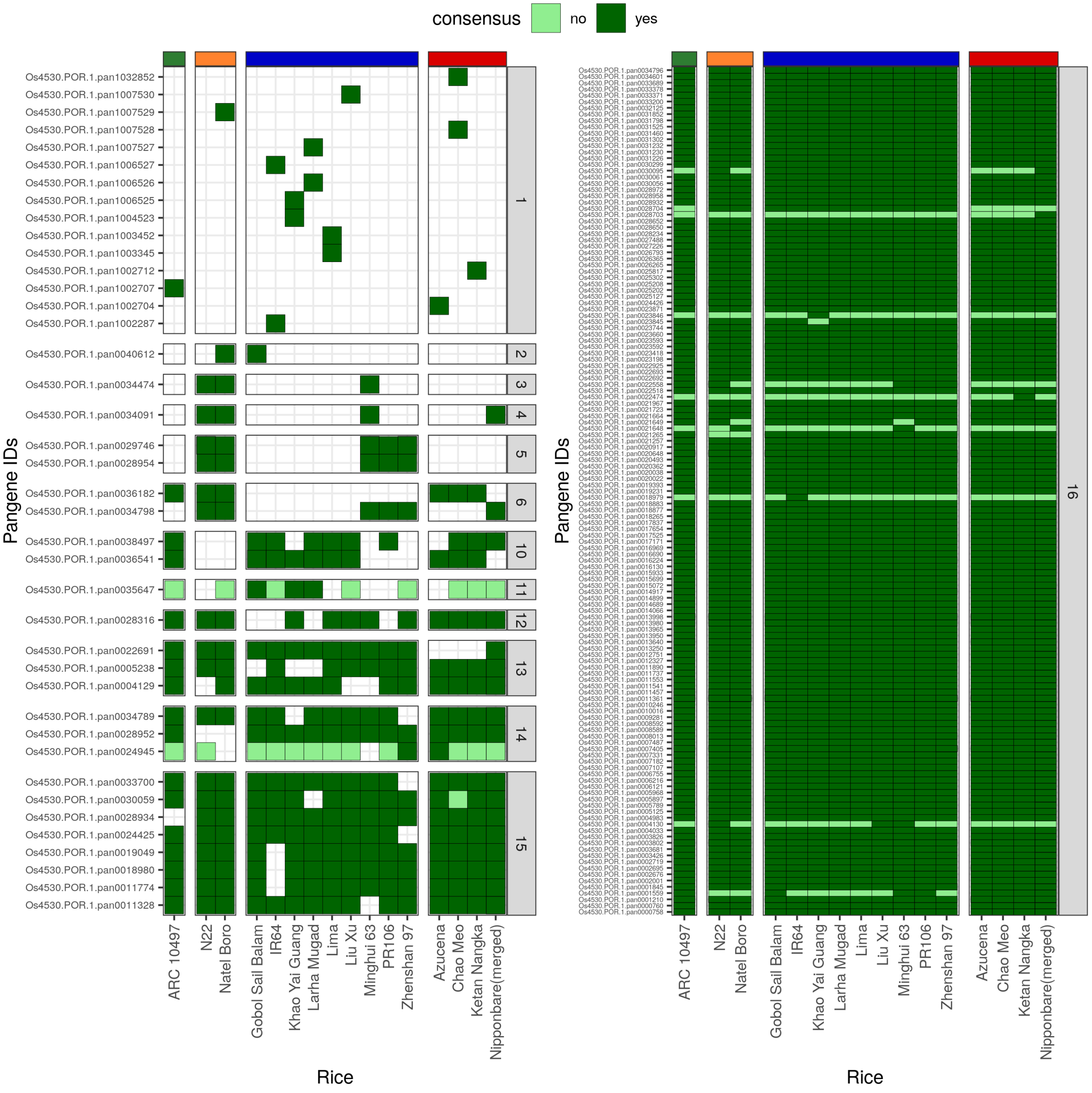

Supplemental figure S6. Pan-genes of NAC transcription factor family. The heatmap shows the pan-genes with at least one member protein containing the NAC domain. The vertical bars coloured grey on the right of heatmap show the occupancy of the pan-gene indicated by a number inside the box. The horizontal bars on top of the heatmap represents the varietal group colored red for *japonica*, blue for *indica*, orange for *aus* and green for *aromatic* rice accessions. The consensus presence /absence of the domain in a family member protein is indicated by dark and light shades of green in the box, respectively, whereas absence of the gene member is indicated by white box.

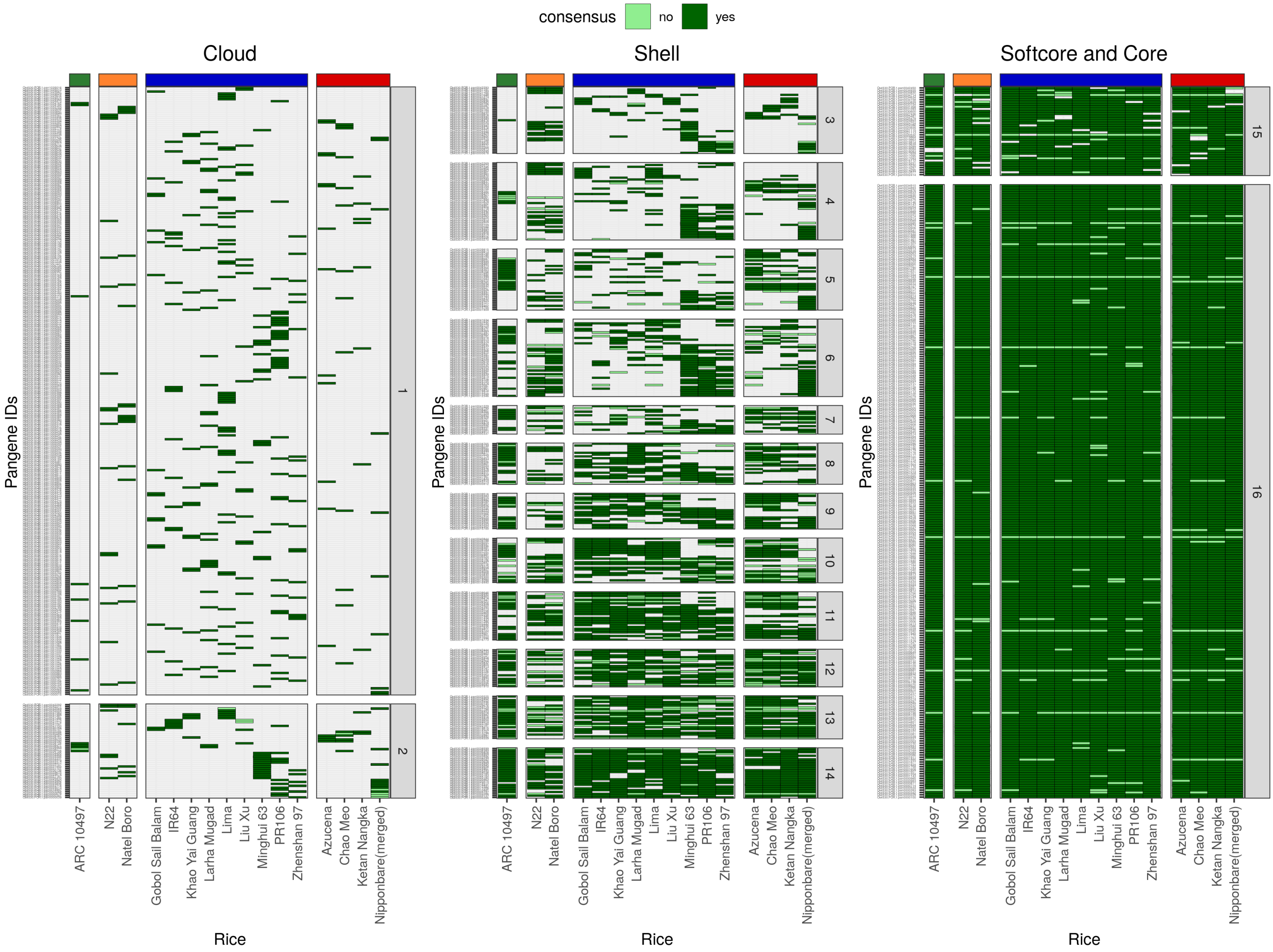

Supplemental figure S7. Pan-genes of NB-ARC family in rice. The heatmaps show pan-genes with at least one member protein containing the NB-ARC domain. The three heatmaps are arranged according to the occupancy classes – cloud, shell and softcore and core. The vertical bars coloured grey on the right of heatmap show the occupancy of the pan-gene indicated by a number inside the box. The horizontal bars on top of the heatmap represents the varietal group colored red for *japonica*, blue for *indica*, orange for *aus* and green for *aromatic* rice accessions. The consensus presence /absence of the domain in a family member protein is indicated by dark and light shades of green in the box, respectively, whereas absence of the gene member is indicated by white box.

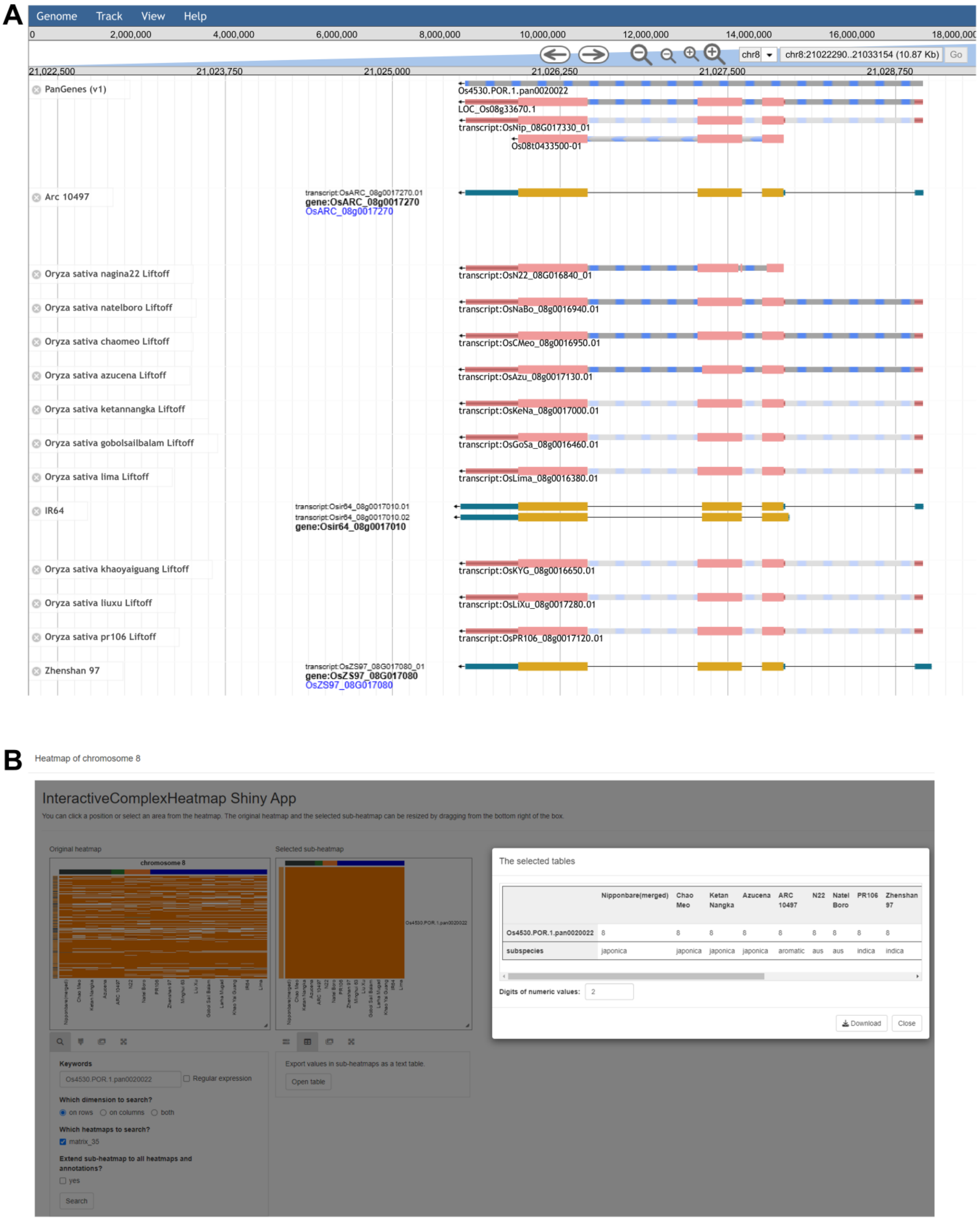

Supplemental figure S8. Representative examples of infrastructure for pan-gene exploration. A) The screen shot shows the record of pan-gene Os4530.POR.1.pan0020022 on the Rice Genome Browser (Jbrowse) hosted on PanOryza project website (https://panoryza.org/). B) The panel shows the an interactive heatmap of chromosome 8 where all members of Os4530.POR.1.pan0020022 are located (detailed on the sub-heatmap and table). The pan-gene interactive heatmap for all chromosomes has been made available as a shiny application (https://github.com/PGB-LIV/PanOryza-pan-genes-release-v1.0/tree/main/heatmap_app).

A

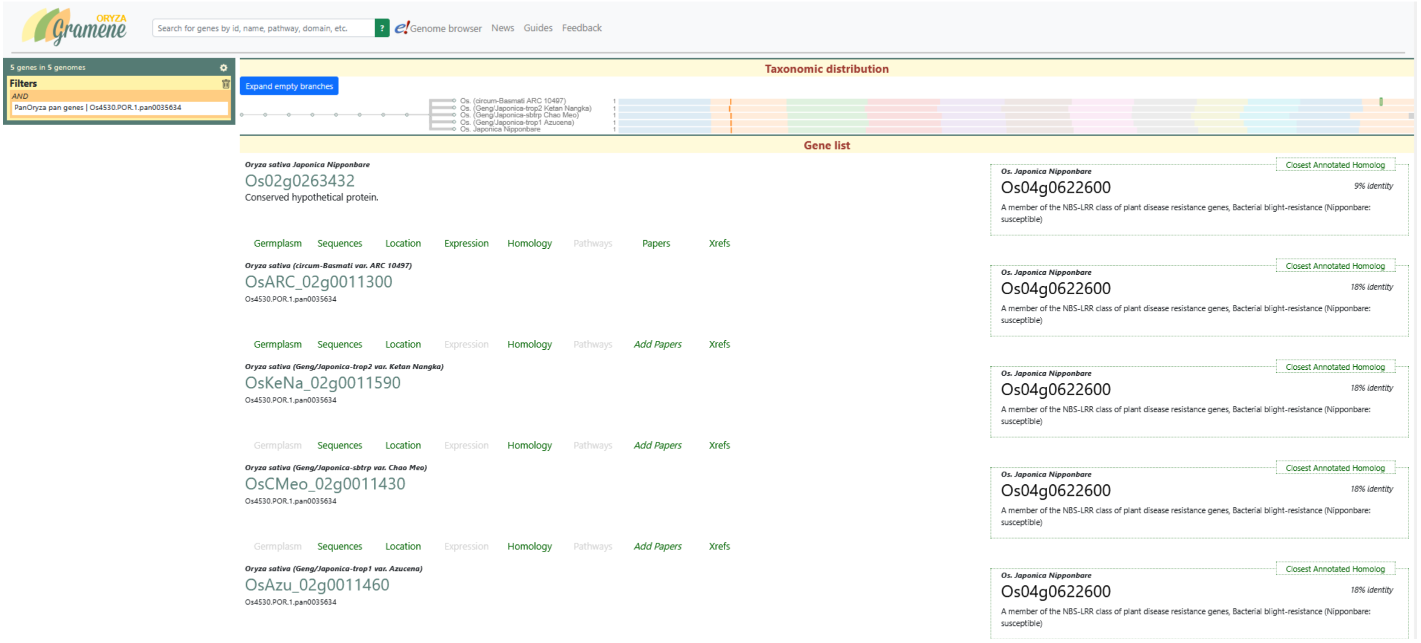

B

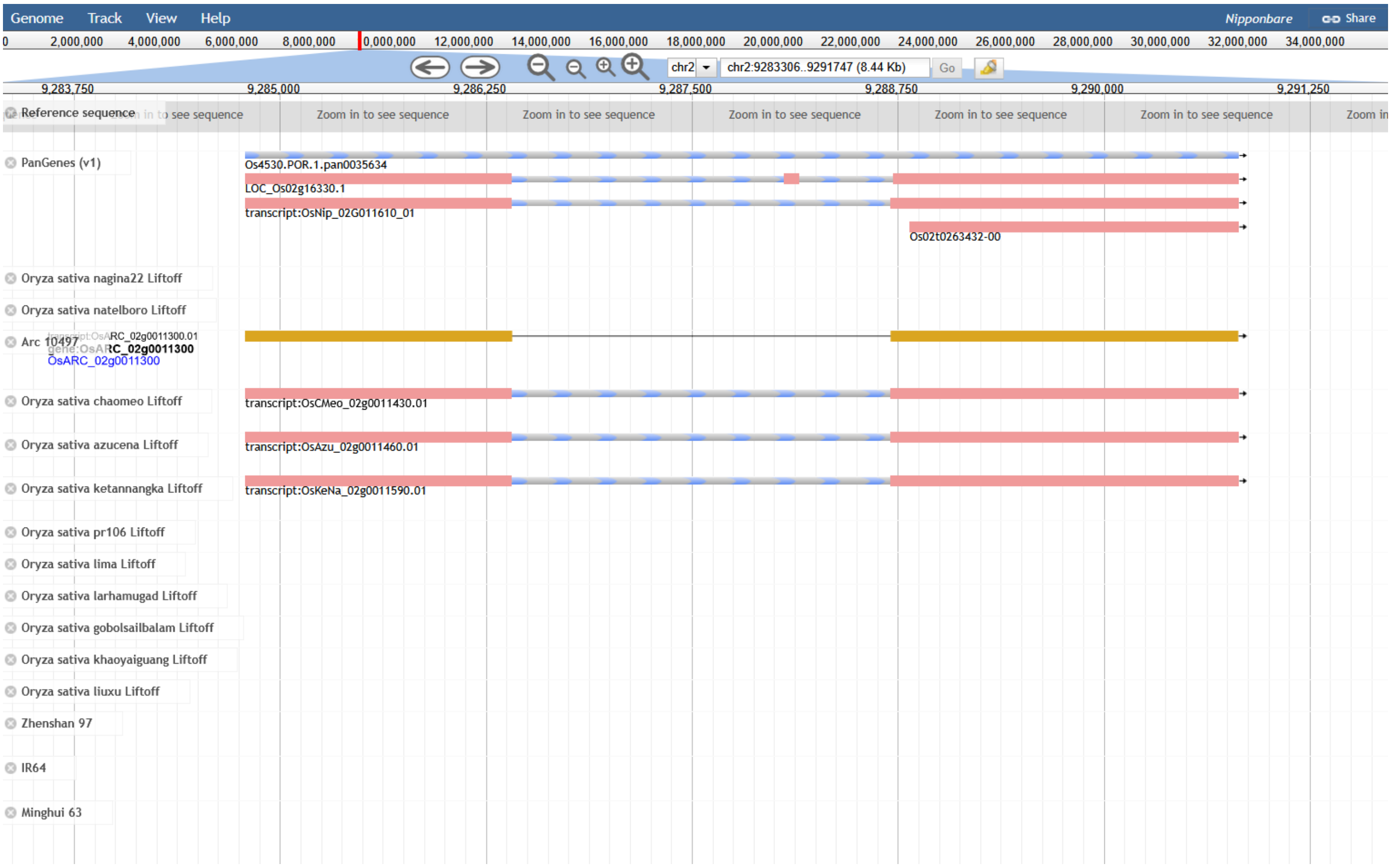

Supplemental figure S9. Representative example pan-gene Os4530.POR.1.pan0035634. A) The screen shot shows the record of pan-gene Os4530.POR.1.pan0035634 on the Gamene (https://oryza.gramene.org). B) The screen shot shows the record of Nipponbare gene Os02g0263432 (Os4530.POR.1.pan0035634) on PanOryza project website (https://panoryza.org/). The gene structure from other RPRP genomes is also shown (wherever gene is present).

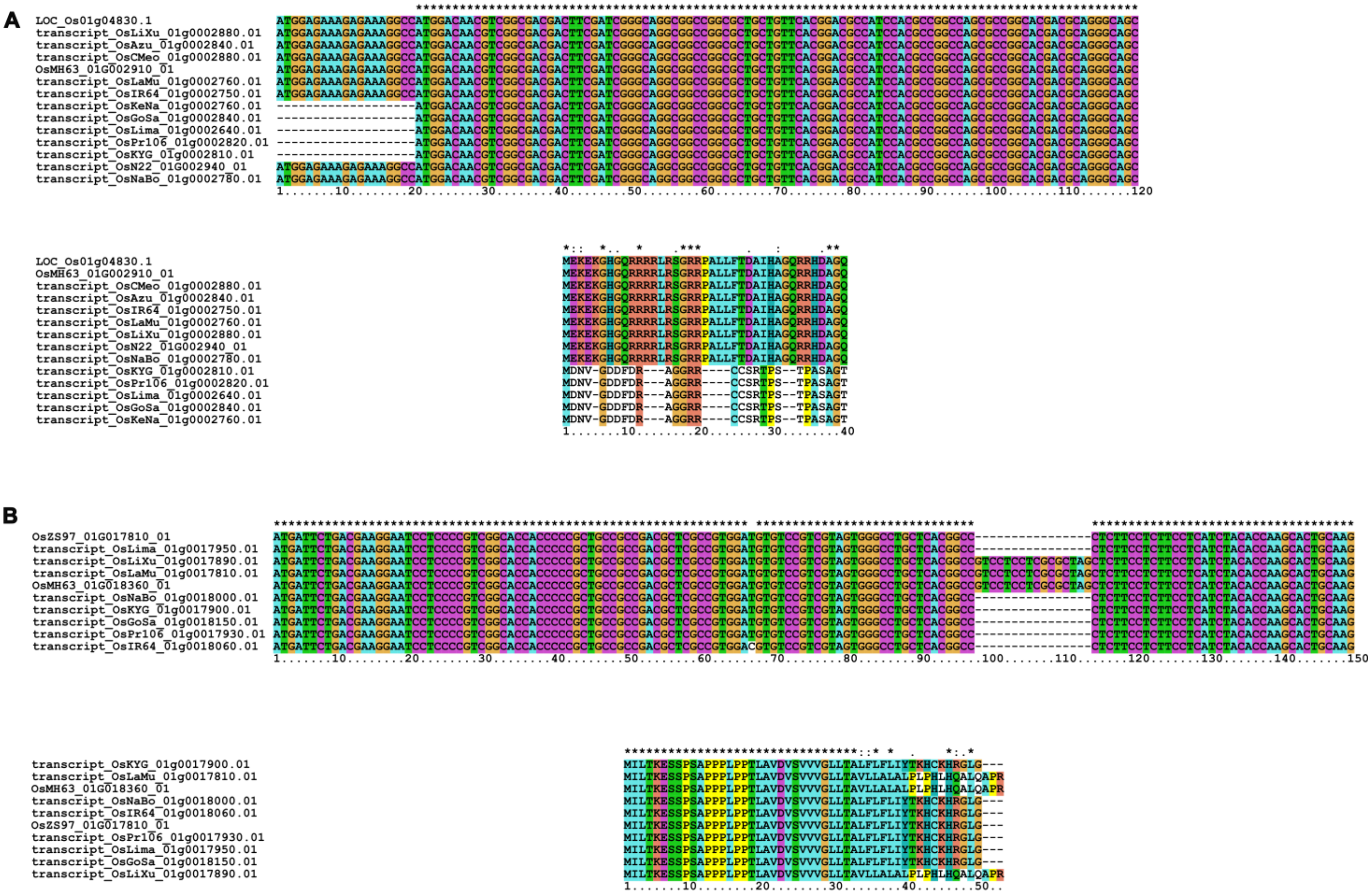

Supplemental figure S10. Multiple alignment of N-terminal CDS sequences encoded by pan-gene A) Os4530.POR.1.pan0000003 and B) Os4530.POR.1.pan0000029. A) Nucleotide alignment of 120 nucleotides. The aligned CDS are identical, with exception of five cases where the upstream start codon (ATG) was replaced by another codon 20 nucleotides downstream on a different frame. B) Nucleotide alignment of 150 nucleotides. The aligned CDS are nearly identical, with exception of three cases with an insertion in position 98 that introduced a frameshift. Alignment of the corresponding amino acid sequences, with the asterisks on the top line indicating the identical aligned sites. Alignments computed with ClustalX 2.1. Given that the conserved frameshift is observed in multiple genomes, this may be evidence of novel function generation or pseudogenization in multiple genomes.

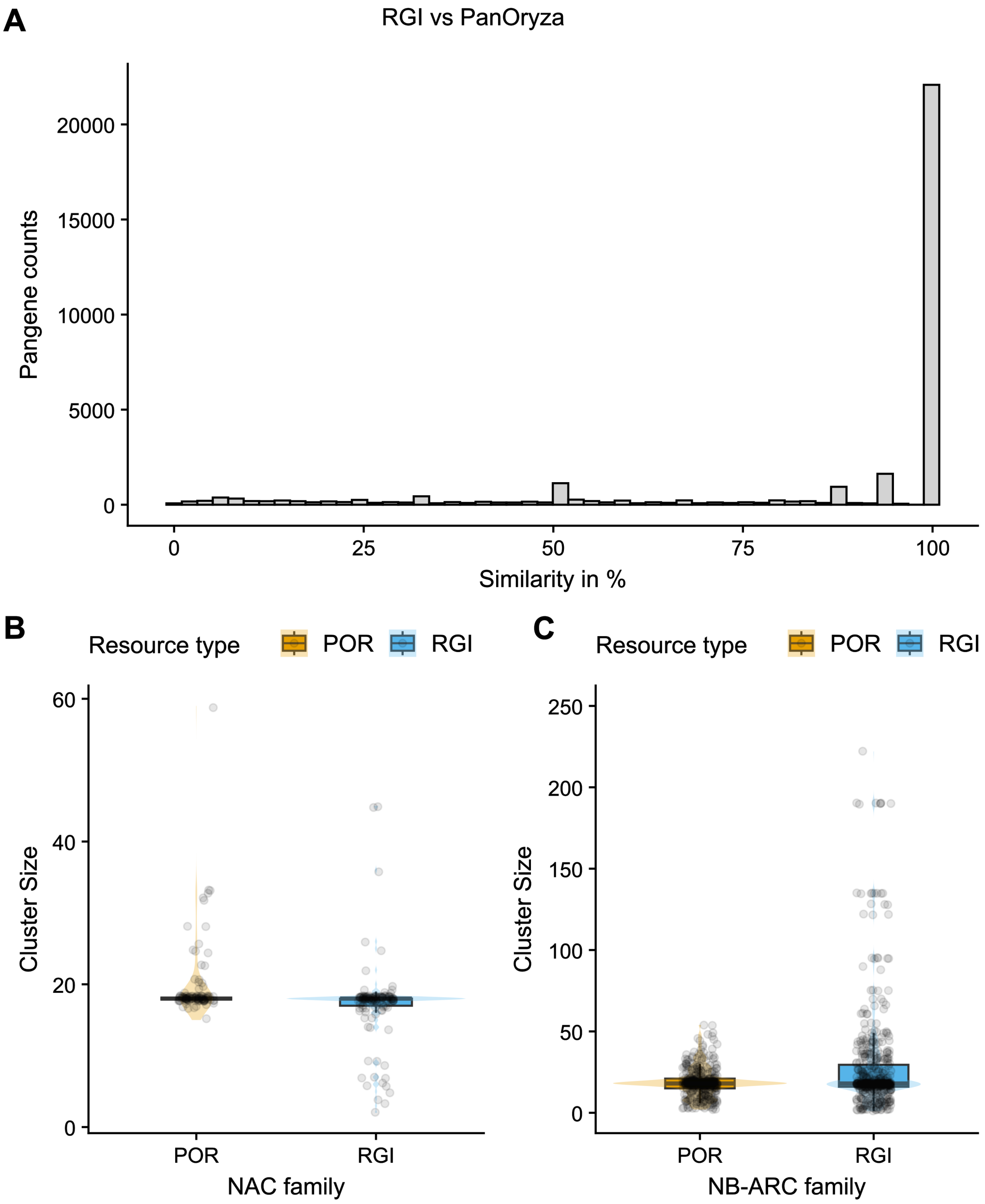

Supplemental figure S11. Comparison of current pan-genes with rice gene index. A) The plot shows percentage agreement between the pan-genes in this work with the OGI clusters from the Rice Gene Index (RGI; excluding Minghui 63 , Zhen Shan 97 and Nipponbare (Gramene) annotations). The percentage agreement of identifiers within each pan-gene is indicated on x-axis and the counts of pan-genes on the y-axis. Comparison of the size of pan-gene clusters between PanOryza (POR; this work) and RGI for the two gene families B) NAC and C) NB-ARC is shown in the plots. The cluster size of each pan-gene is shown as a gray dot.

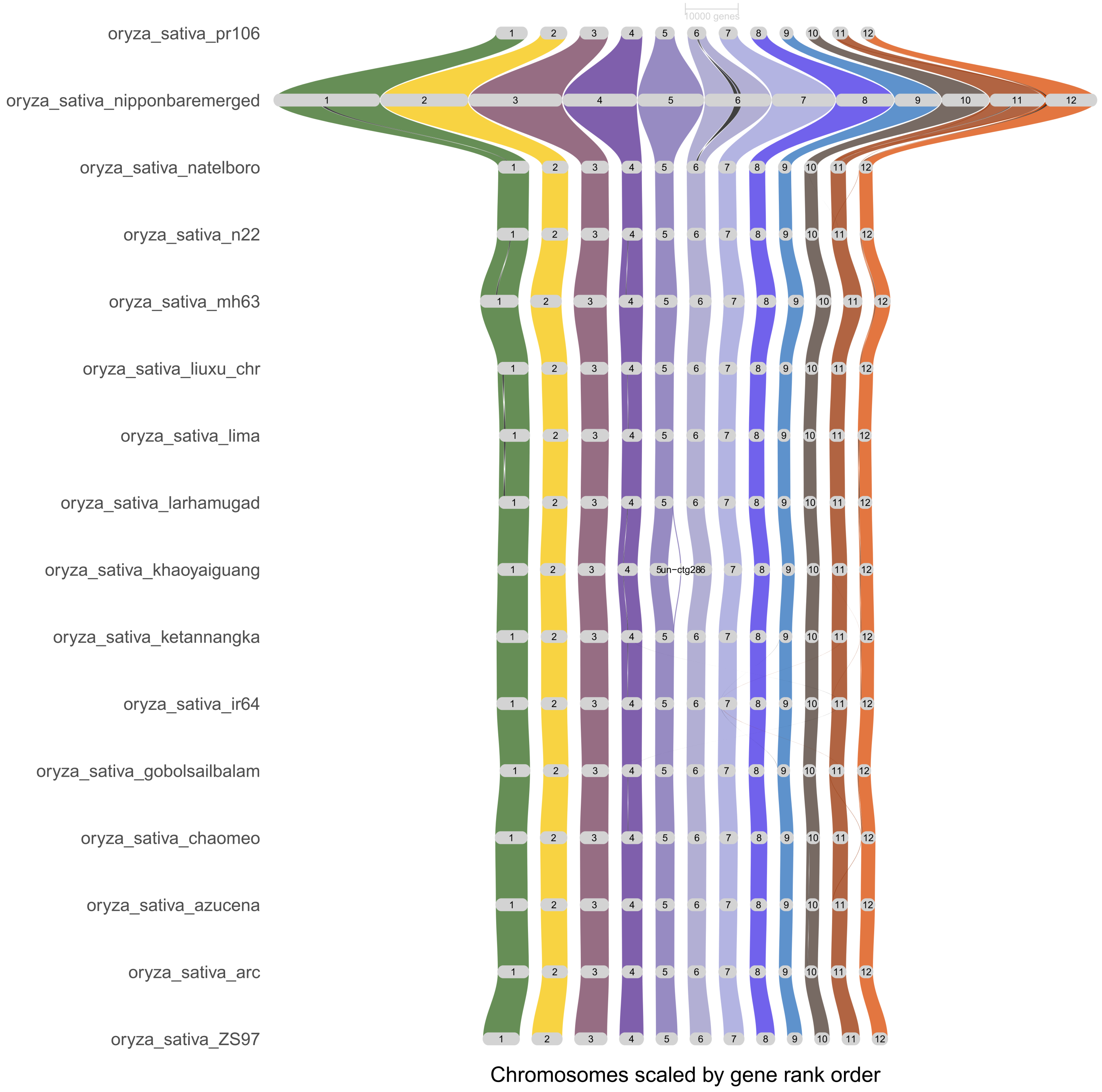

Supplemental figure S12. Riparian plot showing syntenic orthologs of the proteins in the pan-gene clusters based on Nipponbare (merged) and other RPRP accessions. The protein sequences in each pan-gene cluster were deconstructed into individual genomes based on their accession and analysed using GENESPACE algorithm and visualized here using *Oryza sativa* Nipponbare (merged) genome as a reference.

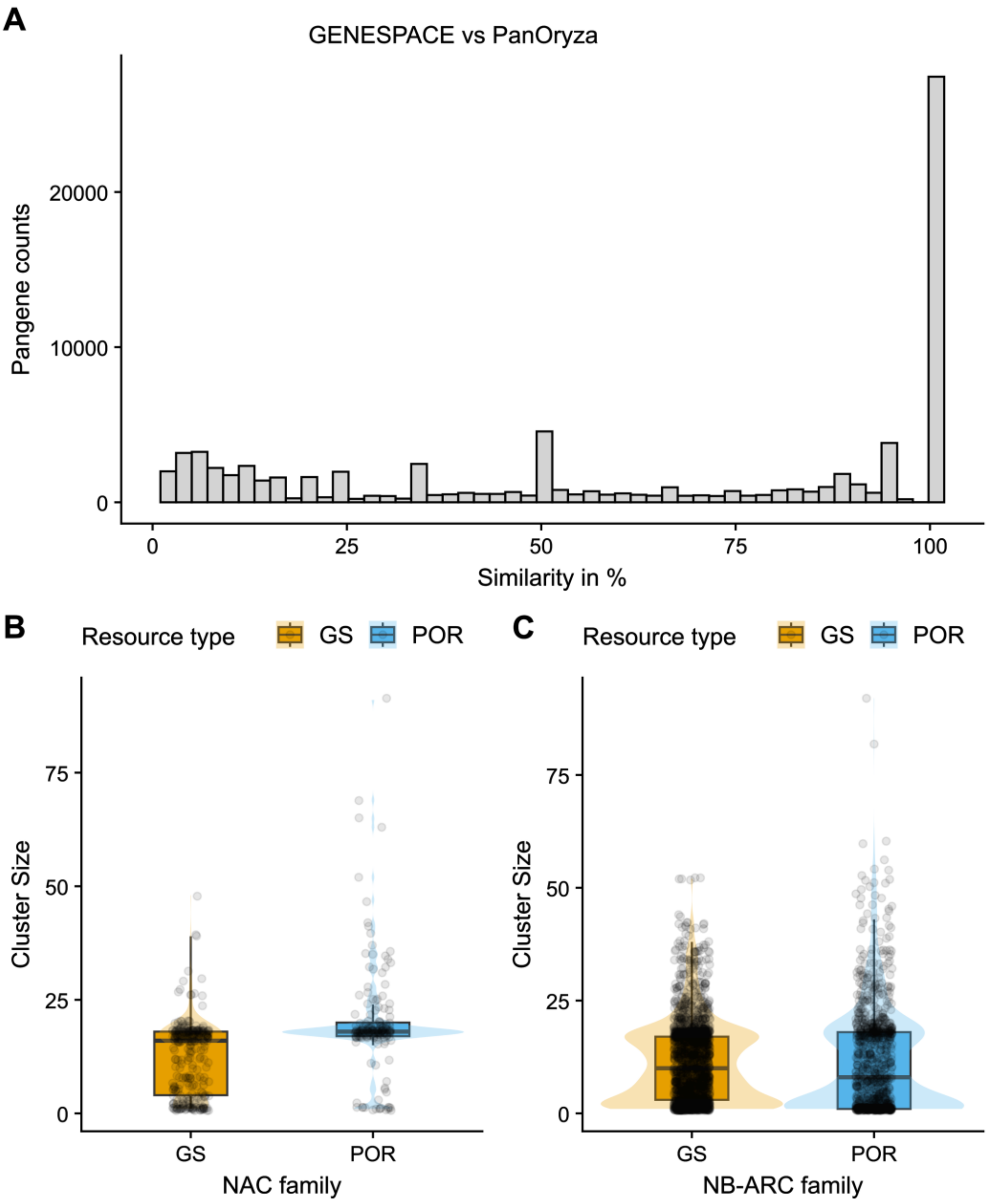

Supplemental figure S13. Comparison of pan-genes identified using GET_PANGENES (current study) vs GENESPACE. A) The plot shows percentage agreement between the pan-genes in this work created using whole genome alignment with the pan-genes identified based on protein sequences using GENESPACE pipeline. The percentage agreement of identifiers within each pan-gene is indicated on x-axis and the counts of pan-genes on the y-axis. Comparison of the size of pan-gene clusters between PanOryza (POR; this work) and those generated using GENESPACE (GP) for the two gene families B) NAC and C) NB-ARC is shown in the plots. The cluster size of each pan-gene is shown as a gray dot. The interquartile range for the NAC cluster size is 3 for PanOryza and 14 for GENESPACE, and GENESPACE assigns 49 singleton genes, compared to only 15 singleton genes with PanOryza, indicative of better ortholog clustering in PanOryza.

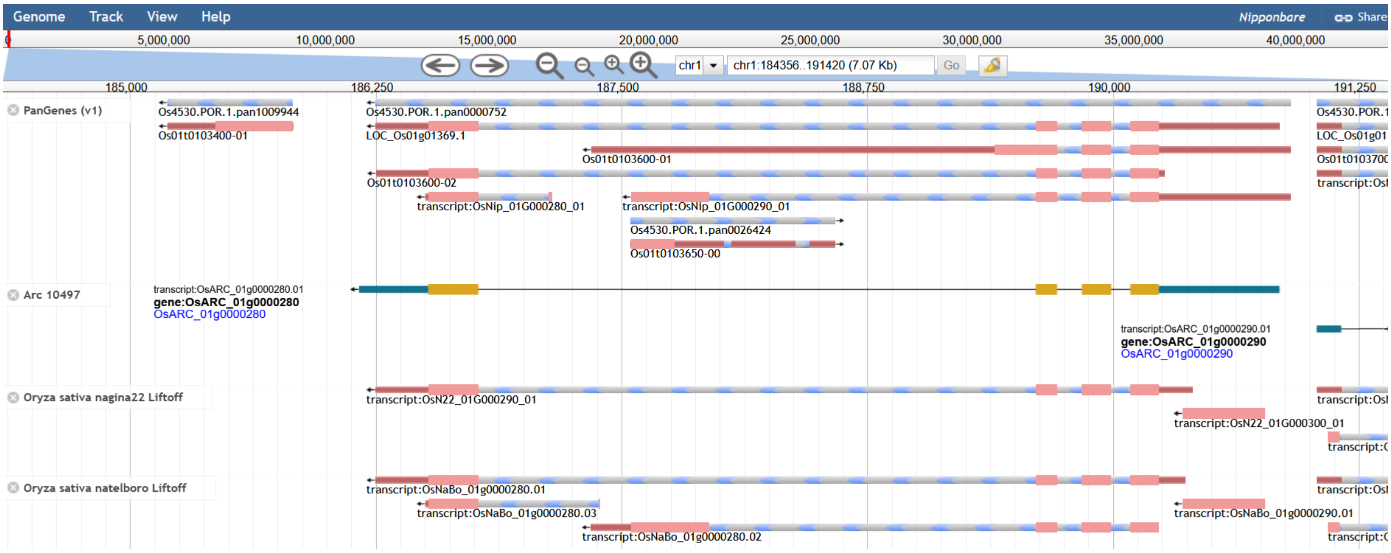

Supplemental figure S14. Example pan-gene Os4530.POR.1.pan0000752 (Os01g0103600). A) Nipponbare gene model with 5 isoforms and 5 exons after merging MSU, RAPDB and IsoSeq annotations. B) IsoSeq-based model of cultivar Arc. C) Two IsoSeq-based models of cultivar Natel Boro, one with 3 isoforms, that split the Nipponbare model in two. D) Two IsoSeq-based models of cultivar N22 that split the Nipponbare model in two.
